# Concurrent jellyfish blooms and tenacibaculosis outbreaks in Northern Norwegian Atlantic salmon (*Salmo salar*) farms

**DOI:** 10.1371/journal.pone.0187476

**Published:** 2017-11-02

**Authors:** Sverre Bang Småge, Øyvind Jakobsen Brevik, Kathleen Frisch, Kuninori Watanabe, Henrik Duesund, Are Nylund

**Affiliations:** 1 Cermaq Group AS, Dronning Eufemiasgate 16, Oslo, Norway; 2 Fish Disease Research Group, Department of Biology, University of Bergen, Thormøhlensgate 55, Bergen, Norway; Stockholm University, SWEDEN

## Abstract

Tenacibaculosis is an increasing problem in the Norwegian Atlantic salmon aquaculture industry causing significant economic losses. In September 2015, two separate outbreaks of suspected tenacibaculosis occurred at two Atlantic salmon farms in Finnmark County in Northern Norway. The events resulted in major losses of smolts newly transferred into seawater. Prior to, and during the outbreaks, large numbers of small jellyfish, identified as *Dipleurosoma typicum* (Boeck) were observed in the vicinity of the farms and inside the net-pens. This study investigates the possible link between the jellyfish, *Tenacibaculum* spp. and the tenacibaculosis outbreaks. Bacteriology, histology, scanning and transmission electron microscopy, and real-time RT-PCR screening were performed on both fish and jellyfish samples. Based on the findings, *Tenacibaculum finnmarkense* was found to be the dominant bacteria associated with the tenacibaculosis outbreaks at both sites and that *D*. *typicum* is unlikely to be a vector for this fish pathogenic bacterium. However, results do show that the jellyfish caused direct damage to the fish’s skin and may have exacerbated the bacterial infection by allowing an entry point for bacteria.

## Introduction

Jellyfish blooms are a rising concern to the marine aquaculture industry because they have been associated with an increasingly large number of mortality events in Atlantic salmon farming, which have resulted in economic losses and fish welfare issues [1,2]. This phenomenon is not fully understood but is possibly due to changing ocean conditions, including anthropogenic causes, and/or that production is now occurring in areas with naturally high occurrences [3–5]. High levels of *Pelagia noctiluca* in Northern Ireland have been linked with several mortality events with Atlantic salmon exhibiting skin and gill lesions, as well as abnormal behaviour such as increased jumping [2,6,7]. This species has also been linked to a mortality event in Atlantic Coastal France [8]. In Scotland and Norway, mass mortality events showing mainly gill lesions have been linked to blooms of *Phialella quadrata* [9,10]. Mortality events in Norway have also been associated with blooms of: *Aurelia aurita*, *Muggiaea atlantica*, *Apolemia uvaria*, and *Bolinopsis infundibulum* [11–13]. Some of these jellyfish (e.g. *Aurelia aurita* and *Pelagia noctiluca*) have been experimentally shown to cause gill and skin damage to marine-farmed fish without the need for other stressors or pathogens [14,15].

The ability of jellyfish (in this paper, members of Phylum Cnidaria are referred to as “jellyfish”) to form blooms under favourable environmental conditions is due to them having both asexual and sexual reproduction [4]. All jellyfish have the potential to be toxic due to having cnidocytes mainly found on the tentacles, which contain stinging nematocysts [16]. These are highly specialised organelles that fire a venom containing structure when triggered in response to a chemical or physical stimuli such as contact with a fish’s skin [17,18]. There are variations in the composition of the venoms between jellyfish species and some have been shown to be cytotoxic or haemolytic [15,19]. The severity of mechanical and toxic injury caused by jellyfish is exacerbated by certain factors such as increased temperatures and exposure [15].

Studies have shown that many environmental bacteria, including pathogenic ones such as *Tenacibaculum* spp. (Family Flavobacteriaceae) and *Moritella viscosa* (Family Moritellaceae) are found on jellyfish and these could therefore act as vectors [10,20–22]. *Tenacibaculum* spp. are found worldwide with some species causing tenacibaculosis in marine aquaculture; a disease mainly characterised by ulcerative lesions, frayed fins and mouth erosion [23–27]. The bacteria have also been linked to gill lesions [28,29]. Tenacibaculosis has been reported with increased frequency in the last few years in the Norwegian salmon farming industry [30,31]. In Norway, affected fish most commonly have mouth erosion, frayed fins (pectoral, pelvic and anal), and tail rot and these lesions have been associated with *Tenacibaculum finnmarkense* [25]. Lesions are often characterised by skin ulcers with yellow margins that are surrounded by wide areas of scale loss (personal observations). Similar lesions have been associated with jellyfish mortality events [6]. The literature would therefore suggest that such mortality events are a result of the direct damage by the jellyfish, as well as associated filamentous bacterial infections [2,13,32].

In September 2015, two separate outbreaks of suspected tenacibaculosis occurred at two Atlantic salmon farms in Finnmark County in Northern Norway. The events resulted in major losses of smolts newly transferred into seawater. Prior to, and during the outbreaks, large numbers of small jellyfish (approximately 10 mm in diameter) were observed in the vicinity of the farms and inside the net-pens. This study investigates the possible link between the jellyfish, *Tenacibaculum* spp. and the tenacibaculosis outbreaks.

## Materials and methods

### Field sampling

#### Jellyfish

**Fig 1:**
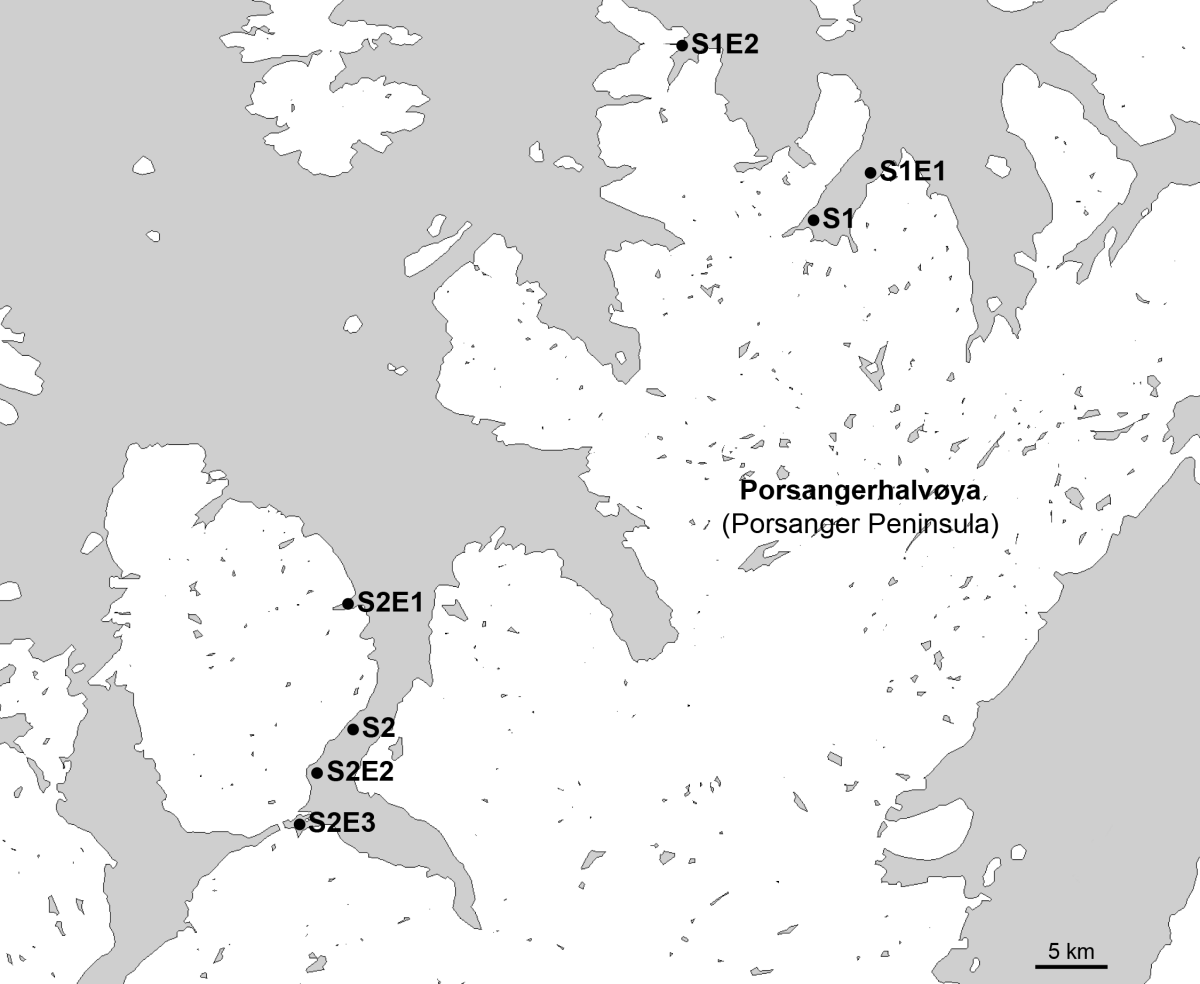
Map of sampling points. Map showing the area surrounding Porsangerhalvøya in Finnmark in the northernmost part of Norway. Farm sites are marked as S1 and S2 and environmental sampling points are marked with the site number, followed by the environmental sampling point number (e.g. S1E2).

Several specimens of the jellyfish were collected at each sampling point as shown in Fig 1 and placed into individual tubes containing 100% ethanol. Some specimens were also placed directly in a modified Karnovsky fixative [33] for histology, Transmission Electron Microscopy (TEM) and Scanning Electron Microscopy (SEM). Single jellyfish specimens were placed onto Marine Kanamycin Agar (MKA) [34] to look into the *Tenacibaculum* community present on the jellyfish as kanamycin is reported to prevent overgrowth by other environmental bacteria [34–37]. Bacterial samples were identified by their site number, followed by their environmental sampling point if not at the site, and then the jellyfish number (E.g. isolate S1E2J3 was sampled from site 1 at environmental sampling point 2 from jellyfish 3).

#### Fish

All fish samples were collected from two licensed (Norwegian Directorate of Fisheries) Atlantic salmon saltwater farms during two outbreaks of tenacibaculosis. Cermaq Norway who runs both farms, gave permission for licensed aqua medicine biologists to collect samples from their sites during the outbreaks. Both commercial sites receive smolts and grow them to market size using best aquaculture practices following the requirements of the Norwegian Aquaculture Act. Randomly sampled fish were euthanized with an overdose (100 mg L^-1^) of benzocaine (Benzoak vet—Europharma). Moribund fish were euthanized with a swift blow to the head to avoid contamination between fish.

Skin, gill, and some eye samples from moribund fish were immediately placed in a modified Karnovsky fixative for histology and SEM. Bacterial samples from the skin lesions of these fish were collected and grown on Marine Agar (Difco 2216) (MA) and identified by their sample point (Fig 1) followed by the fish number (E.g. isolate S1F3 was sampled from site 1 from fish 3). Skin tissue (margin of lesion and if not present, the skin under the mandible) were collected for *Tenacibaculum* spp. and *M*. *viscosa* real-time RT-PCR screening from 10 moribund fish from affected pens at site 1 and 30 randomly caught ‘healthy’ fish and 5 moribund fish from each net-pen at site 2 (Fig 1).

### Jellyfish identification

One jellyfish specimen from each farm site was removed from the ethanol and lysed in a mixture of OB Protease solution and TL buffer (OMEGA bio-tek) prior to DNA extraction. The DNA was extracted once with an equal volume of phenol/chloroform/isoamyl alcohol (24:25:1), then once with chloroform (24:1), followed by precipitation using 1:10 volume of 3M Sodium Acetate (pH 5.5) and two volumes of ice cold 100% ethanol. The quality and concentration of the eluted DNA was measured by using a Nanodrop (Thermo Scientific) spectrophotometer. For use in PCR reactions, the eluted DNA was diluted using nuclease free water to a concentration of approximately 50 ng μl^-1^. The DNA was stored at -20°C.

PCR of the jellyfish mitochondrial large ribosomal subunit (MLRS) DNA sequence was performed using the forward and reverse primers described in Cunningham and Buss [38]. The amplification was performed using the standard reaction mixture described in Småge, Frisch [39] at 94°C for 5 min, 35 cycles of 95°C for 30 s, 56°C for 30 s, and 72°C for 1 min, followed by 72°C for 10 min in a Veriti Thermal Cycler (Thermo Fisher Scientific). The PCR product was confirmed using gel electrophoresis and then enzymatically purified using ExoSAP-IT PCR Product Cleanup (Thermo Fisher Scientific) in a Veriti Thermal Cycler at 37°C for 15 min followed by 80°C for 15 min. Sequencing was performed on PCR products using the internal primer described in Cunningham and Buss [38]. The analysis was carried out by the Sequencing Facility at the University of Bergen (http://www.uib.no/seqlab) using Big Dye termination chemistry. Obtained sequences were uploaded to The Basic Local Alignment Search Tool (BLAST) (NCBI) to determine the identity of the jellyfish.

A morphological identification of the sampled jellyfish was also performed using the identification keys and notes for identification of thecate hydroids and their medusae [40] and the description given by Russell [41].

### Bacteriology

All primary cultures were incubated for a minimum of 5 days at 16°C and sub-cultivation was performed on MA and incubated at 16°C for 48–72 hours. Bacteria were identified by uploading their DNA sequences to the BLAST. In order to do so, genomic DNA was obtained by heating single colonies obtained from bacterial clones in tubes containing nuclease free water at 95°C for 5 min. The samples were then centrifuged at 10,000 rpm for 5 min and the DNA-containing supernatant was transferred to new tubes and stored at -20°C. PCR was performed using the 16S rRNA primers 27F and 1518R [42] and the amplification and sequencing were performed as described in Småge, Frisch [39].

### Jellyfish real-time RT-PCR analysis

The ethanol in which the jellyfish samples were preserved was removed by centrifuging the tubes at 8,000 rpm for 5 min before discarding the ethanol. Cultured *Halobacterium salinarum* DSM 3754^T^ cells suspended in PBS were added to each of the tubes prior to RNA extraction as an exogenous control in the real-time RT-PCR analysis [43]. The jellyfish samples were then homogenized by adding 1ml of Isol-RNA Lysis Reagent (5 Prime) to each sample and then placed in a TissueLyser LT (Qiagen) at 50 Hz for 5 min followed by 5 min sitting at room temperature. The RNA was extracted following the manufacturer’s protocol (5 Prime), except that a second washing step using 100% ethanol was performed prior to air drying of the RNA pellet. The obtained RNA was subsequently diluted 1:10 in order to avoid inhibition in the real-time RT-PCR reaction and stored at -20°C.

The extracted RNA was tested for the presence of *Tenacibaculum* spp. commonly recovered from tenacibaculosis outbreaks in Norwegian farmed Atlantic salmon using the Tb tuf real-time RT-PCR assay (Table 1) [44]. The exogenous control (*H*. *salinarum*) was detected using the assay (SAL) developed by Andersen, Hodneland [43] (Table 1). All assays were run using an AgPath-ID kit (Thermo Scientific) with 2 μl of RNA and the standard AgPath-ID concentrations of primers (400 nM) and probe (120nM). Each run consisted of 45 cycles.

**Table 1:** Real-time RT-PCR assays.

Assay	Assay Efficacy	Primer/Probe	Sequence
Tb tuf	1.9397	Forward	AGTGTGACGTCCACCTT
		Reverse	CTGTAAGCCAGGTTCTGT
		Probe	TTTCAATACATACACCTCAGC
Mv ompA	1.8826	Forward	GATGATAACGCAACAGCAG
		Reverse	CGGAAACTTACACCAGATAATG
		Probe	TCTTGGAGCAGGTCTAGAATATACACCAG
SAL	1.8501	Forward	GGGAAATCTGTCCGCTTAACG
		Reverse	CCGGTCCCAAGCTGAACA
		Probe	AGGCGTCCAGCGGA
ELF1A	1.9800	Forward	CCCCTCCAGGACGTTTACAAA
		Reverse	CACACGGCCCACAGGTACA
		Probe	ATCGGTGGTATTGGAAC

The sequences of the primers and probes and the efficacy of each real-time RT-PCR assay used in this study [43–45].

### Atlantic salmon real-time RT-PCR analysis

The RNA from the site 1 samples was extracted using Isol-RNA Lysis Reagent (5 Prime) following the manufacturer’s protocol and the RNA from the site 2 samples was extracted by a commercial laboratory. The RNA was screened using real-time RT-PCR with the Tb tuf assay, an assay targeting *M*. *viscosa*: Mv ompA, and an assay targeting the elongation factor 1 alpha (EF1A) [45] (Table 1). All assays were run using the same procedure as for the jellyfish real-time RT-PCR analysis.

### Phylogenetic analysis

Sequences were assembled and aligned using Vector NTI v.9.0 (Invitrogen) software and adjusted to equal lengths using GeneDoc [46]. The alignment included all 16S rRNA gene sequences obtained in this study and for all known type strains in genus *Tenacibaculum*. In addition, the 16S rRNA gene sequences of *Flexibacter echinicida* and *Flexibacter aurantiacus subsp*. *copepodarum*^T^ [47] were included to improve the phylogenetic resolution. The bacterium *Kordia algicida*^T^ AB681152 was used as an outgroup as suggested by Habib, Houel [48]. All sequences not from this study were obtained from the GenBank. The phylogenetic relationship was calculated using BEAST 1.8 [49] using the best fitted model, a relaxed lognormal molecular clock and a mcmc of 150,000,000 generations. The GTR+G+I model was found to be the best fitted model by using Mega6 [50]. All ESS values were above 200 for the Bayesian analysis and a maximum clade credibility tree was created using a 10% burn-in in Tree-Annotator [49] and viewed using FigTree v1.4.0 [49].

### Histology, scanning and transmission electron microscopy

Preparation of fish and jellyfish tissues for histology and SEM was performed as described in Småge, Frisch [39]. Jellyfish samples for TEM were prepared as described in Nylund, Hovland [33]. Ten jellyfish were prepared for histological examination: five were pre-embedded in 0.4% agarose before embedding in EMbed 812 resin and five were directly embedded in the EMbed 812 resin.

## Results

### Field sampling

At site 1, the tenacibaculosis outbreak started at the beginning of September. Five of the six net-pens were newly stocked with smolts, one to three weeks prior, and had an average weight of 87 grams at the start of the outbreak. The sixth net-pen had been stocked six weeks prior, and the fish were significantly larger (average of 280 grams) at the time of the outbreak. The accumulated mortality in that pen was 2.1% for the duration of the event. The other five pens had to be culled due to fish welfare reasons as a result of the disease.

At site 2 there were two separately stocked smolt populations: six net-pens were stocked at the start of summer with spring smolts and the other six net-pens were stocked at the end of the summer with autumn smolts. In the middle of September, the spring smolts started showing signs of tenacibaculosis followed by the newly transferred autumn smolts. At this point the average weight was 155 grams for the spring smolts and 68 grams for the autumn smolts. Mortality spiked towards the end of the month and continued into the middle of October. All net-pens were treated with florfenicol at 10 mg kg^-1^ for 10 days. The accumulated mortality for the outbreak differed between net-pens and generations. The accumulative mortality per net-pen for the tenacibaculosis outbreak, which lasted approximately one month, was 2.5 to 8% for the spring smolts and 17 to 40% for the autumn smolts.

The water temperatures measured at five meters at both farm sites were between 8 and 10°C at time of sampling. Sampling occurred at the start of the outbreak for both sites. At site 2, three of the net-pens containing spring smolts had already been treated with florfenicol at 10 mg kg^-1^ for 10 days.

**Fig 2:**
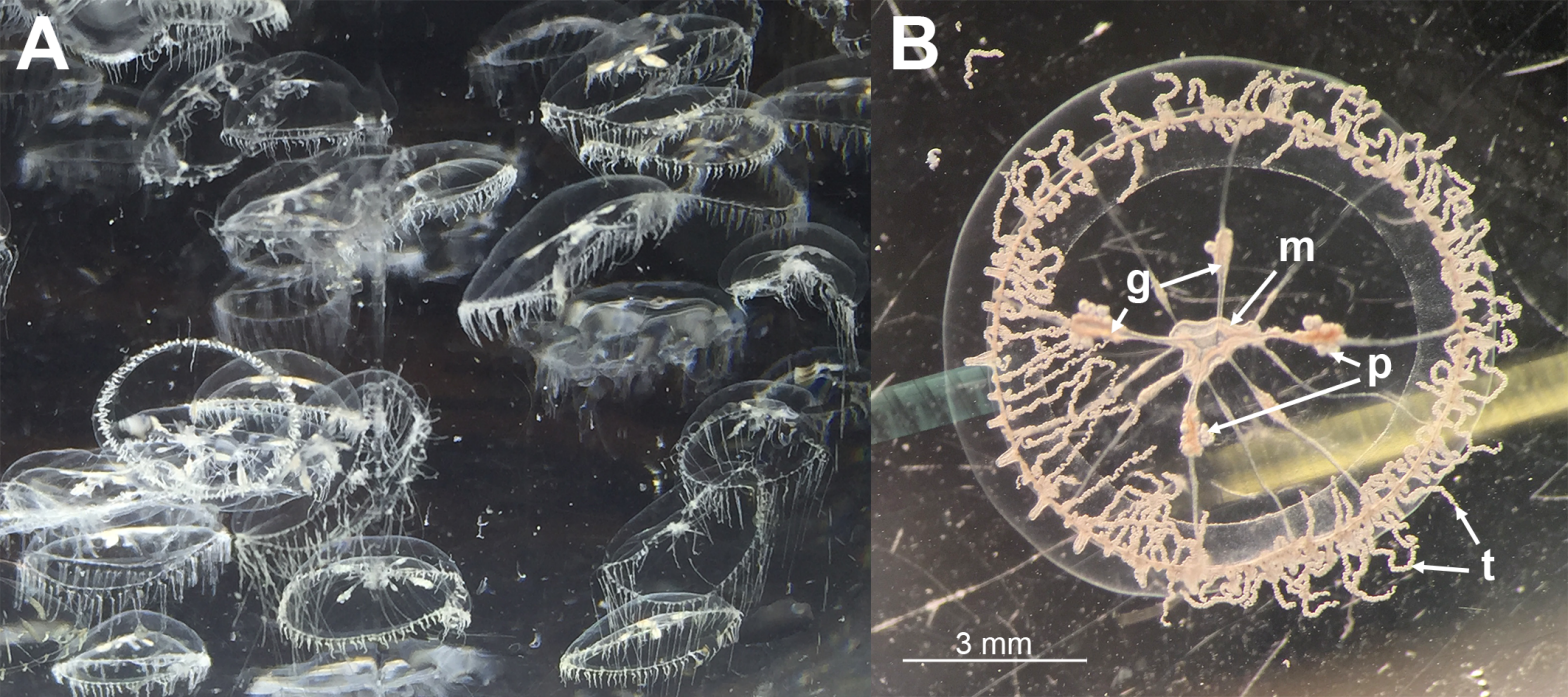
Jellyfish morphology. (A) A water sample collected outside one of the net-pens at site 1. A high concentration of *D*. *typicum* was present in the sample. (B) One of these specimens under a stereomicroscope showing the characteristics typical for this species [40,41]. m: the mouth, t: tentacles, g: gonads, p: planula larva (Note: the planula larva was found to be ciliated and highly motile, which differs from previous descriptions given for this species).

**Fig 3:**
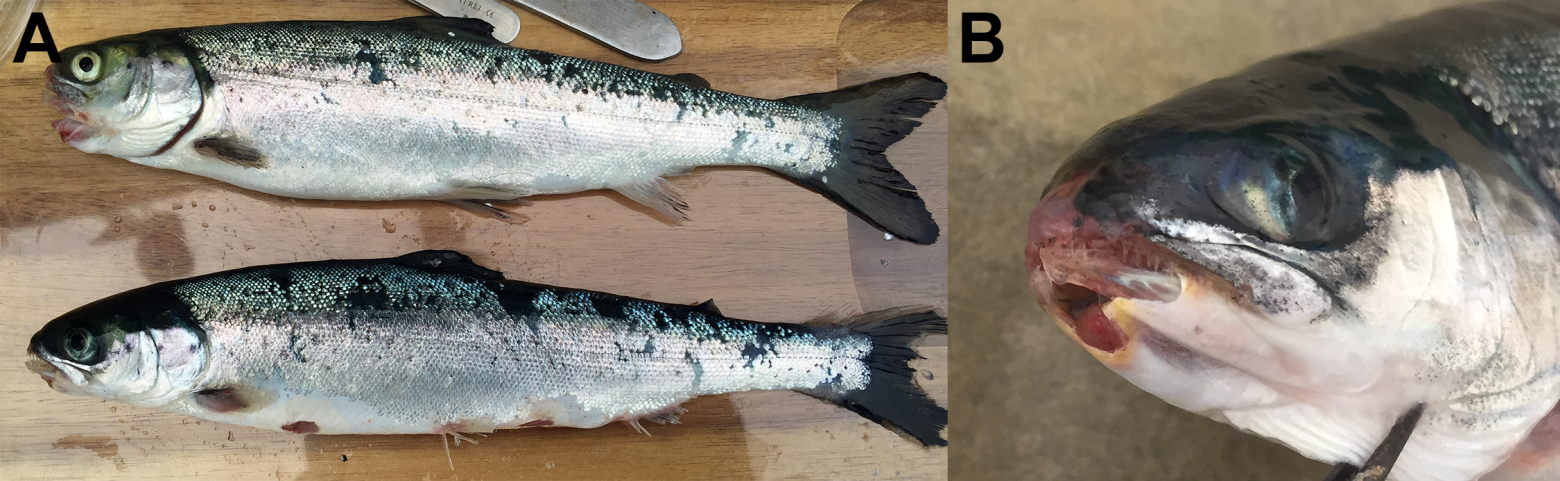
Moribund fish showing signs of tenacibaculosis. (A) Fish representing moribunds from both site 1 and 2. Skin lesions have the typical scale loss and yellow margins seen with tenacibaculosis. Frayed fins are also clearly visible. (B) A close up of an affected head showing a mouth lesion that has the typical yellow margin associated with tenacibaculosis.

During the sampling, large blooms of small (approximately 10 mm) transparent jellyfish (Fig 2) were present in and around the net-pens at both sites, as well as throughout the surrounding fjords. In certain areas aggregations of the jellyfish gave a greyish cloudy appearance to the sea. The individual jellyfish were found to be very friable thus hampering the sampling. Fish in the net-pens showed abnormal behaviour, including swimming high in the water column and increased jumping activity. Moribund fish showed signs of tenacibaculosis: frayed fins, scale loss, and skin ulcers and mouth erosion with yellow pigmentation of the margins (Fig 3). Some fish had eyes with a cloudy appearance. No gross pathology was observed internally.

Large numbers of small pollock (*Pollachius virens*) were observed feeding on the jellyfish at both sites. One of these fish was caught and found to have jellyfish remains in the stomach on necropsy.

### Jellyfish identification

Based on the morphological description, the dominating jellyfish found at both sites were leptomedusae of *Dipleurosoma typicum* [51] (Phylum Cnidaria; Class Hydrozoa; Subclass Hydroidolina; Order Leptothecata; Family Dipleurosomatidae). The BLAST search of the MLRS DNA sequences did not yield any matches as there are no sequences of this organism in the database; therefore, the identification of the jellyfish had to be purely based on the morphological features. Both macroscopic (Fig 2) and microscopic examinations of the sampled jellyfish showed the features classically described for *D*. *typicum* (i.e. size, number of radial canals, and the position and characteristics of the tentacles and gonads). The obtained *D*. *typicum* sequences from site 1 and site 2 were identical to each other except for one nucleotide. These sequences have been made available in the GenBank (accession number: MF192883 from site 1 and MF192884 from site 2).

### Bacteriology

**Fig 4:**
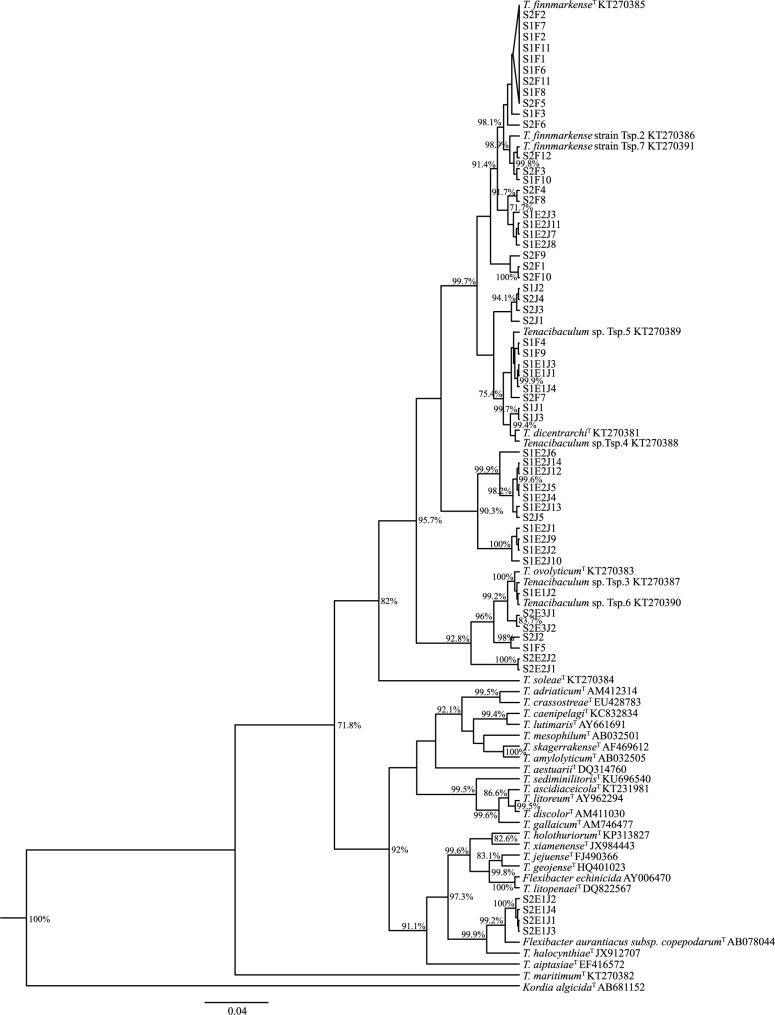
Phylogenetic analysis of recovered *Tenacibaculum* spp. isolates. Phylogenetic tree of the 16S rRNA gene sequences obtained in this study and all known type strains in genus *Tenacibaculum*. In addition, *Flexibacter echinicida* and *Flexibacter aurantiacus subsp*. *copepodarum*^T^ were included. A Bayesian analysis was performed using the GTR+G+I model.

Bacterial cultures from the fish revealed mostly yellow colonies containing filamentous bacteria that were identified based on the obtained 16S rRNA gene sequence to be *Tenacibaculum finnmarkense*^T^ or closely related strains (Fig 4). The bacteria recovered from the jellyfish revealed a wide range of Flavobacteriaceae: *Tenacibaculum* spp. (Fig 4), *Winogradskyella* sp., *Lacinutix* sp. *Krokinobacter* sp., *Polaribacter* sp., *Olleya sp*. and *Cellulophaga algicola*. *Tenacibaculum* spp. were only recovered from a small number of the jellyfish sampled. No *M*. *viscosa* was recovered from the fish or from the jellyfish.

### Jellyfish real-time RT-PCR analysis

A low number of jellyfish were positive to *Tenacibaculum* spp. with real-time RT-PCR analysis, which was reflected in the bacteriology results. However, the initial results showed inhibition, determined by the exogenous control; therefore the analysis was repeated by diluting the RNA 1:10. The resulting Ct values were close to the detection limit.

### Fish real-time RT-PCR analysis

**Fig 5:**
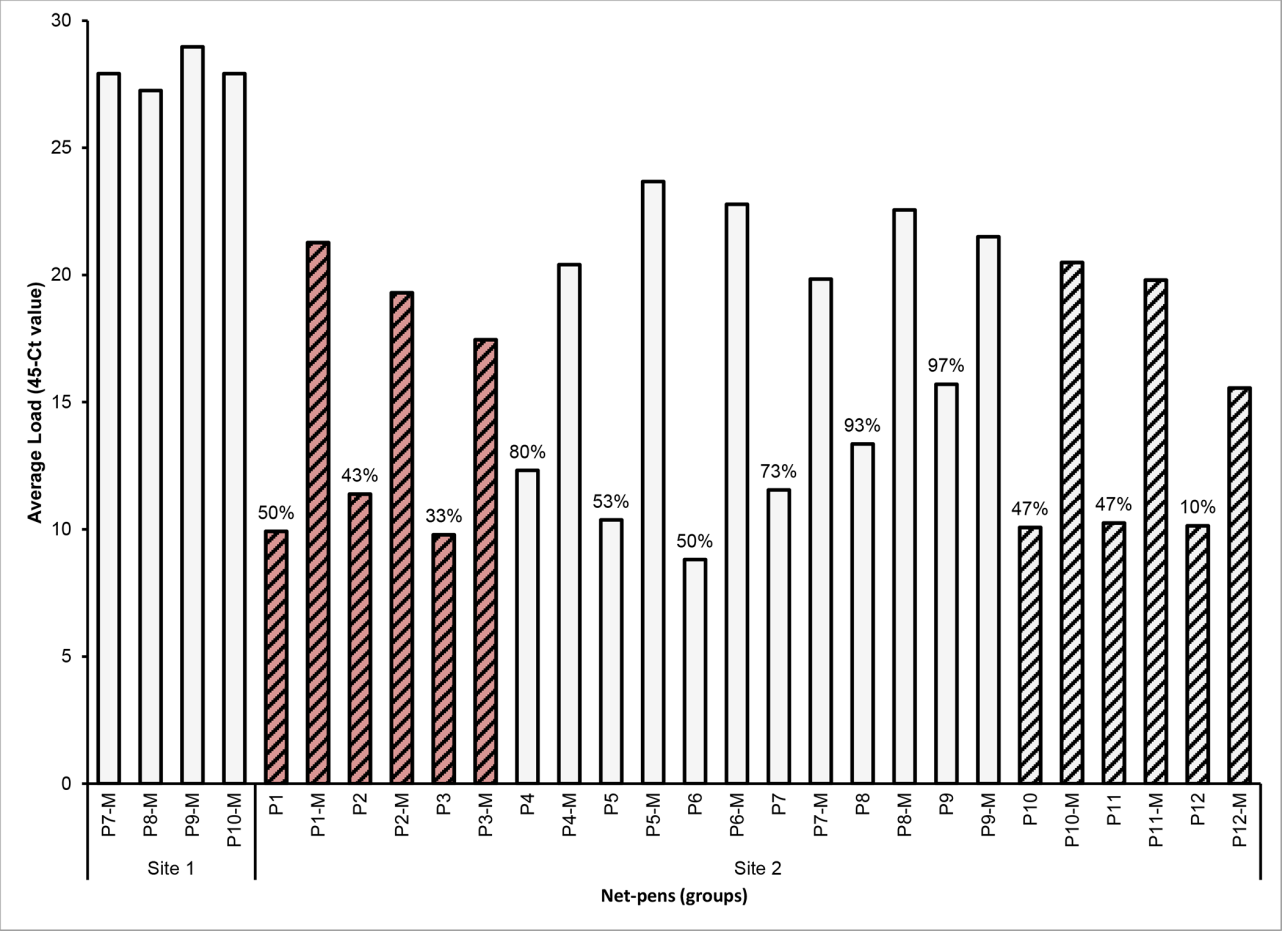
Real-time RT-PCR results from sampled fish. Average load of *Tenacibaculum* spp. positive fish for each sampled group measured with the Tb tuf real-time RT-PCR assay. Dataset includes both randomly sampled fish from each pen at site 2 (n = 30) and moribunds (-M) from each affected pen (n = 10 for site 1 and n = 5 for site 2). The prevalence in the moribunds was 100% and the prevalence in the randomly sampled fish is indicated as % above the respective bar. Striped columns are spring smolts and non-striped columns are autumn smolts. Red columns were treated with florfenicol prior to sampling.

All sampled moribund fish at both site 1 and 2 were positive with the Tb tuf assay indicating that they are positive for *Tenacibaculum* spp. Only two of these fish were positive for *M*. *viscosa* and both had high Ct values indicating a very low load. Of the randomly sampled fish from site 2, none were positive for *M*. *viscosa* and only a proportion of them were positive for *Tenacibaculum* spp. using the Tb tuf assay as indicated in Fig 5. The average load for the moribund fish was higher than that of the randomly sampled fish (Fig 5).

The RNA obtained from the randomly sampled fish, determined by the EF1A results, was within the acceptable quality range. However, some of the samples from the moribund fish were outside this quality range. The loss of quality is most likely due to sampling lesions with a high probability of containing necrotic and therefore degraded tissue. For this reason normalized Ct values were not used for analysis of results in this study.

### Phylogenetic analysis

A total of 57 *Tenacibaculum* isolates were recovered in this study: 23 from fish and 34 from jellyfish (GenBank accession numbers: MF192899 to MF192955). The phylogenetic analysis (Fig 4) showed that the majority of isolates recovered from fish were identical to the type strain or other strains of *T*. *finnmarkense*. Three isolates were more closely related to *Tenacibaculum dicentrarchi*^T^ and one isolate was more closely related to *Tenacibaculum ovolyticum*^T^. The recovered *Tenacibaculum* isolates from the *D*. *typicum* samples revealed a greater phylogenetic diversity than seen in the isolates recovered from the fish; however, none were shown to be identical to the type strain of *T*. *finnmarkense*. A small group of jellyfish isolates were closely related to *T*. *ovolyticum*^T^ and another group were closely related to *Flexibacter aurantiacus subsp*. *copepodarum*^T^.

### Histology, SEM, and TEM

#### Jellyfish

**Fig 6:**
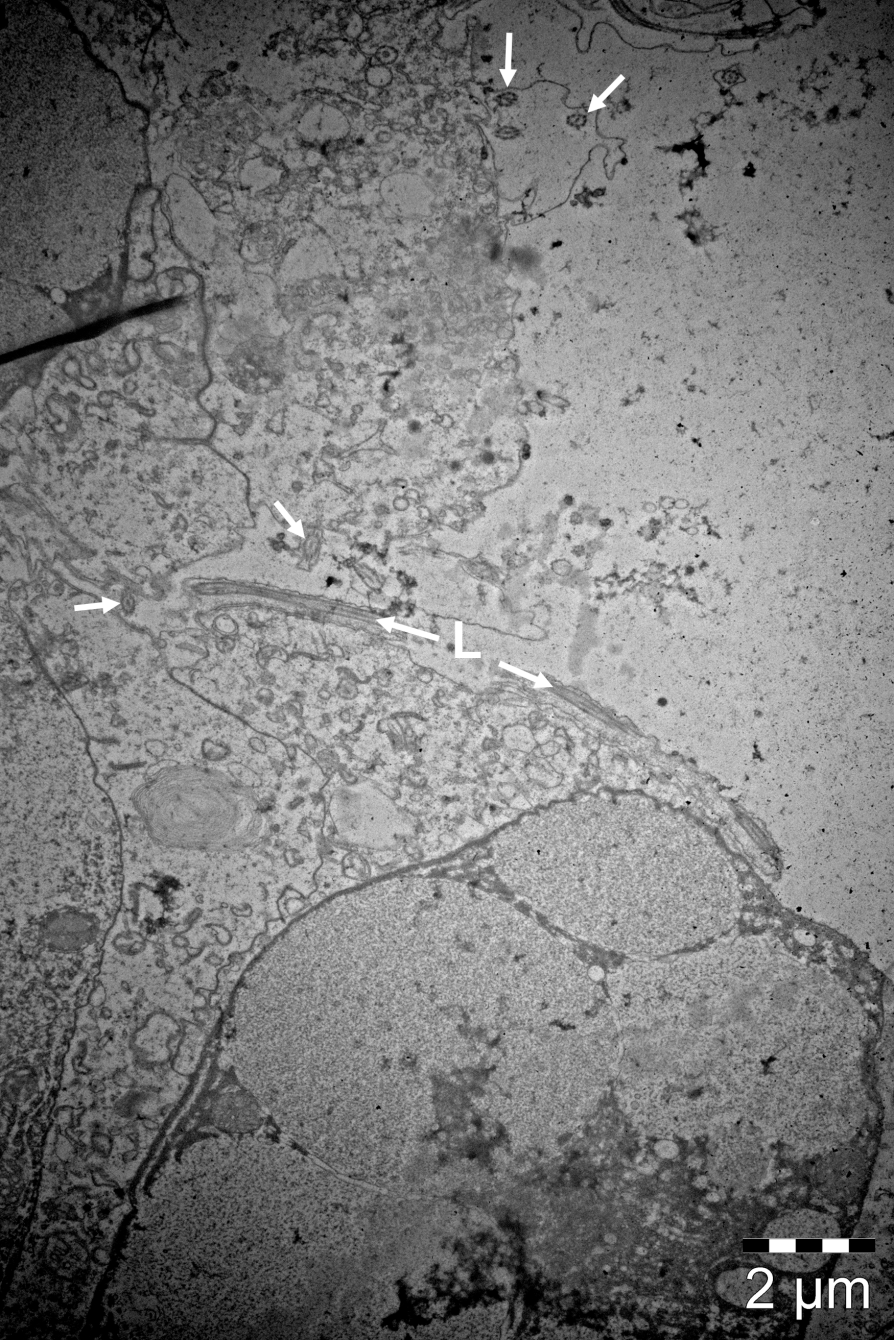
TEM micrograph of the mouth region of *D*. *typicum*. The typical 9+2 pattern (arrows) of motile ciliary axonemes are clearly visible. A cilium can be seen longitudinally (L).

**Fig 7:**
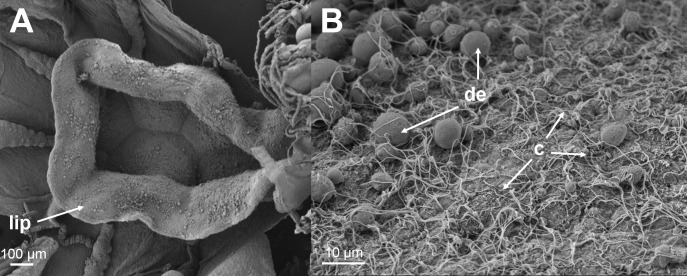
SEM micrographs of *D*. *typicum* mouth region. (A) A SEM micrograph showing the lip of the mouth of *D*. *typicum*. (B) A higher magnification of the inside of the mouth showing an abundance of cilia (c) and spherical structures that are likely droplets of exoenzymes (de) important in the digestion of food [10].

No bacteria were observed on the histologically examined jellyfish. Thread-like structures were observed on the surface of the mouth region on histology, TEM (Fig 6) and SEM (Fig 7) sections that were identified to be surface cilia based on the descriptions given by Fisch and Dupuis-Williams [52], Mariscal [53] and Martin and Archer [54]. Structures were observed on both histology and TEM sections that correspond to what is described for nematocysts. These structures were observed in the epidermis of the mouth and in abundance in the tentacle regions.

#### Fish

**Fig 8:**
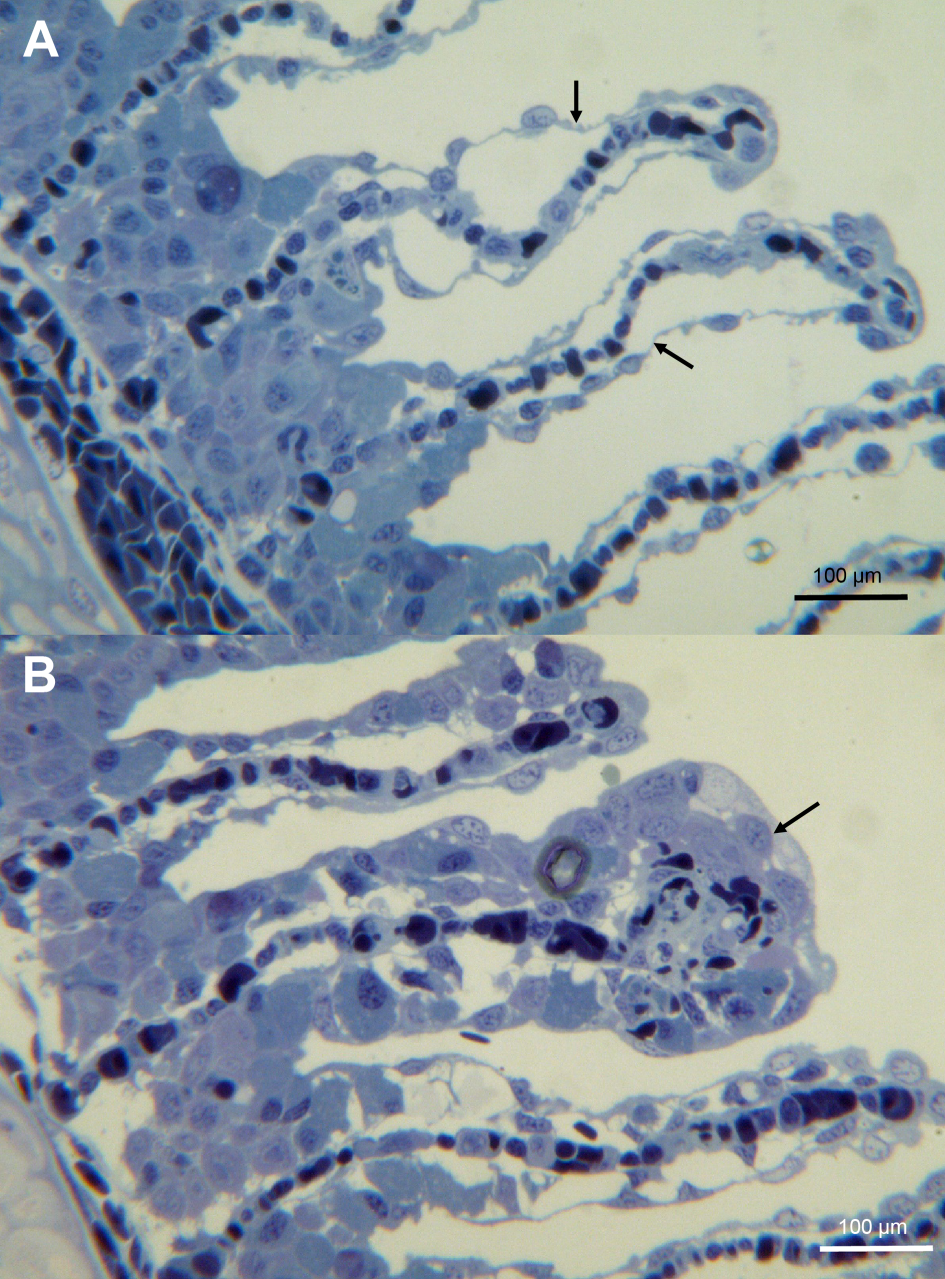
Histology of gills from moribund fish. (A) A toluidine blue stained section of gills showing epithelial lifting (arrow). (B) A toluidine blue stained section of gills showing foci of hypertrophy (arrow).

On histology, sampled gills showed little tissue changes with the exception of some lamellae that had epithelial lifting (Fig 8A) with a few foci of hypertrophy and necrosis (Fig 8B). SEM examination of the gills revealed that the microridges were intact and no nematocysts or pathogens were found (S1 Fig). Filamentous bacteria were seen on the surface of some sampled eyes during SEM examination; however there was no associated tissue damage with the exception of a few nemocysts penetrating the cornea (S2 Fig).

**Fig 9:**
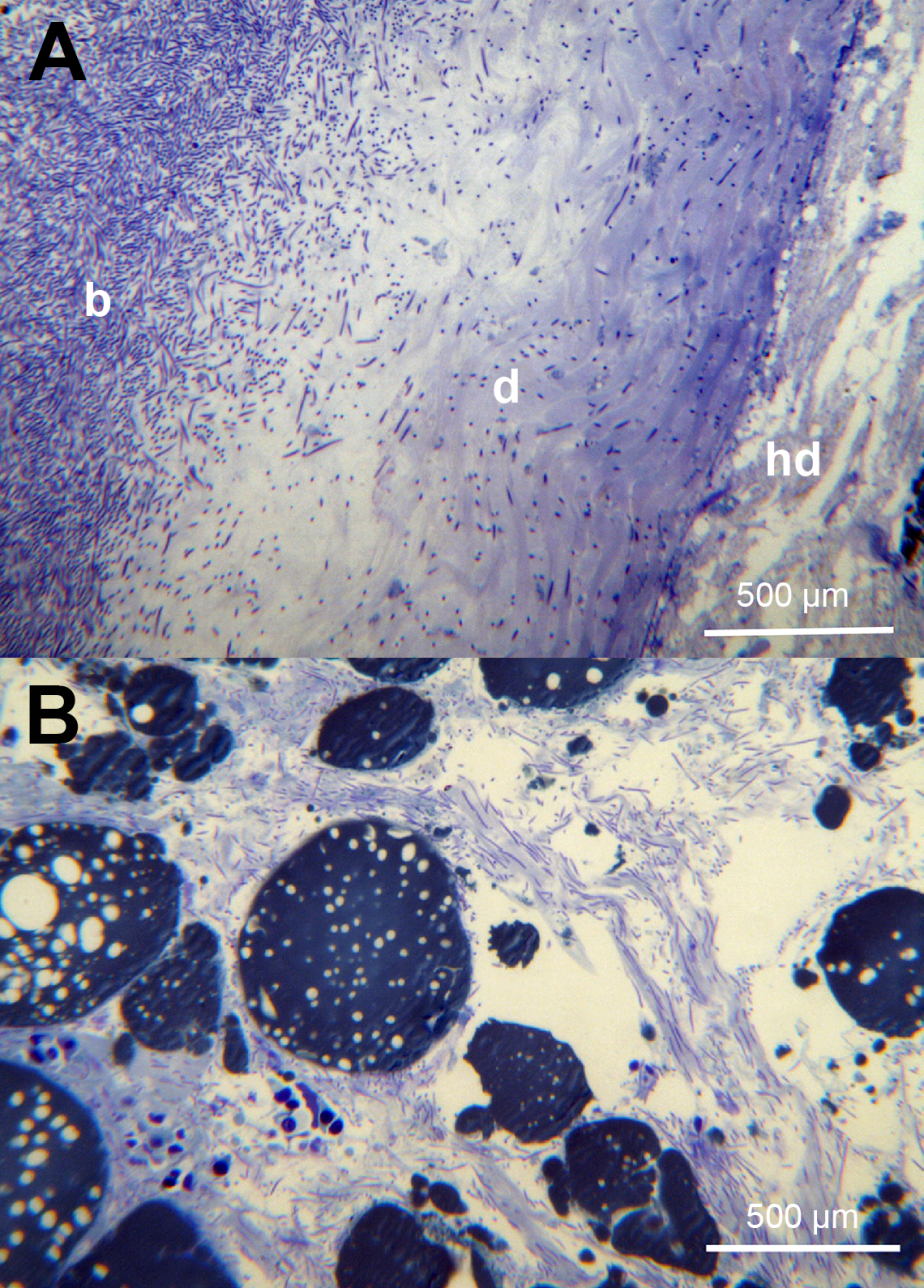
Histology of skin from moribund fish. (A) A toluidine blue stained section of skin showing complete loss of epidermis where the bacteria (b) are present and infiltration of the stratum compactum of the dermis (d). This section does not appear to have bacteria infiltrating the hypodermis (hd). (B) A toluidine blue stained section of the skin hypodermis showing large number of bacteria.

**Fig 10:**
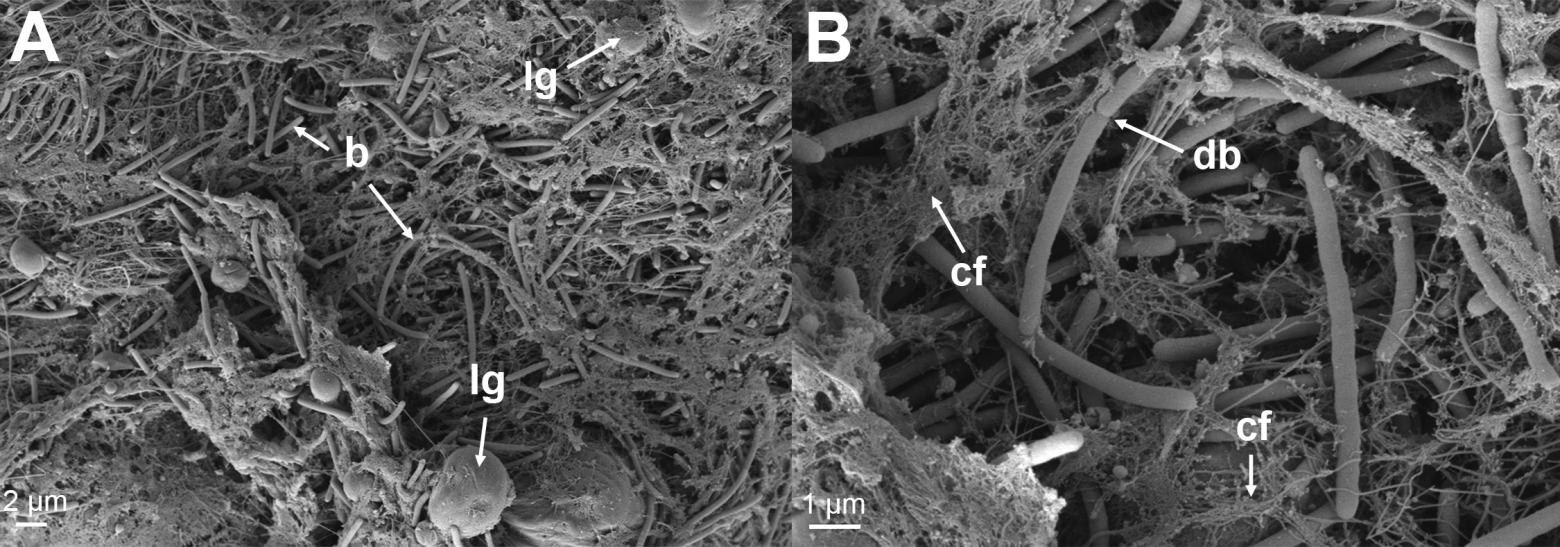
SEM micrographs of lesions from moribund fish. (A) A SEM micrograph from the margin of a jaw lesion showing *Tenacibaculum*-like bacteria (b) infiltrating the dermis layer of the skin. Lipids globules (lg) can be seen and are likely a result of the destruction of the dermis layer as seen histologically. (B) A higher magnification showing a *Tenacibaculum*-like bacterial cell replicating in the dermis (db). The breakdown of the collagen fibers (cf) of the dermis can be seen associated with the bacteria.

**Fig 11:**
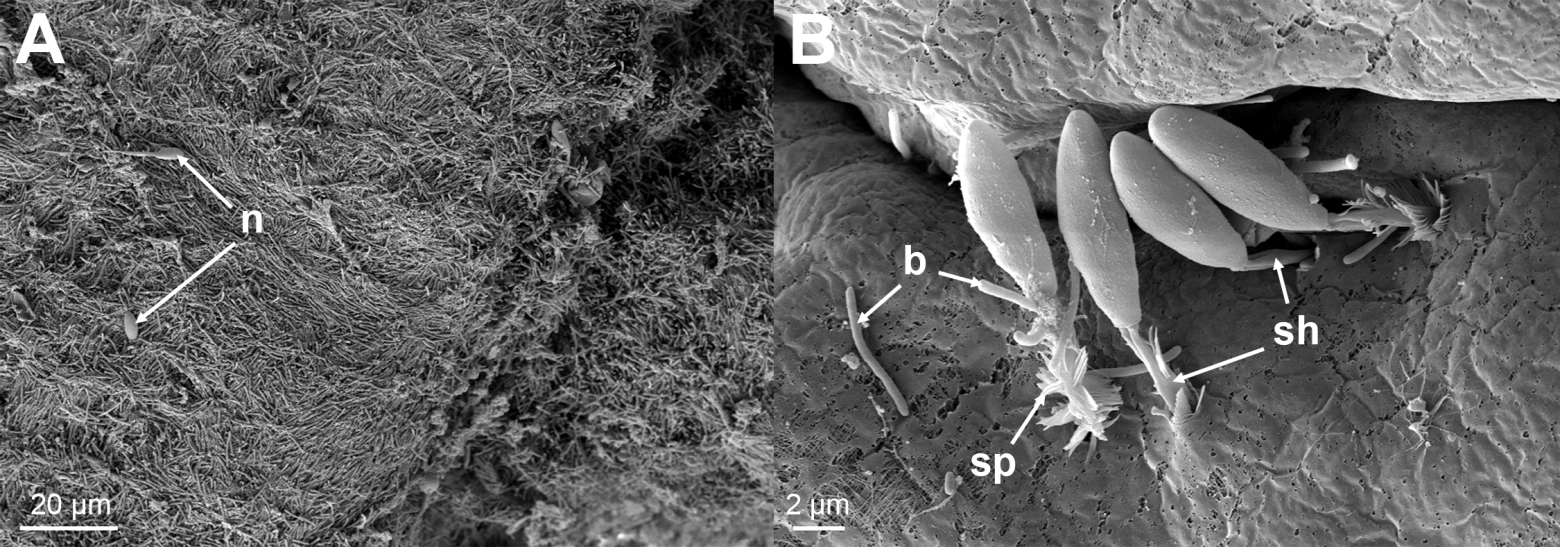
SEM micrographs. (A) A SEM micrograph from the margin of a lesion showing a mat of *Tenacibaculum*-like bacteria with a few attached nematocysts (n). (B) A higher magnification of one of these areas showing the nematocysts with their spiked (sp) covered shafts (sh) embedded in the skin. Bacteria (b) can be seen in close proximity with some seeming to be entering the holes created by the nematocysts.

The skin from all of the sampled fish revealed significant damage to the tissue associated with large numbers of *Tenacibaculum*-like bacteria (Fig 9 and Fig 10), that looked like mats in some areas. The bacteria were not only present in areas of epidermis loss, but were also seen infiltrating the dermis causing degradation of the stratum compactum (Fig 9A). In a few cases the bacteria had penetrated all the way down to the hypodermis and caused damaged to the connective tissue (Fig 9B). Bacterial cells could be seen to be in the process of dividing and were closely entangled with the damaged collagen layer (Fig 10B). Nematocysts were observed on some of the sections from these fish and were seen to be embedded in the skin (Fig 11B) accompanied by large numbers of *Tenacibaculum*-like bacteria (Fig 11A). Holes, the same size as the nematocysts’ shafts were observed, and are likely a result of them being ripped out (S3 Fig).

## Discussion

This is the first case study of a tenacibaculosis outbreak in Atlantic salmon in Norway. Based on the results, the authors suspect that the tenacibaculosis was primarily caused by *T*. *finnmarkense*, but exacerbated by the presence of large amounts of jellyfish. The phylogenetic analysis showed that 19 of the 23 isolates recovered from diseased fish belonged to the *T*. *finnmarkense* clade and approximately half were identical to the type strain. The dominance of this species during both outbreaks strongly supports it being the causative agent of tenacibaculosis in Atlantic salmon in Northern Norway. The very low prevalence of *M*. *viscosa* detected in this study (0.4%) is similar to another published case of ulcerative disease in Northern Norway [55]. Therefore, *M*. *viscosa* does not seem to have an important role in causing disease in these particular cases.

Fish behaviour (lethargy, swimming close to the surface and excessive jumping) during the jellyfish bloom matched what is described in the literature [6,9]. Unlike other described jellyfish blooms, the fish showed few gill lesions both grossly and microscopically leading to the conclusion that the fish did not directly die from their exposure [14,15]. The affected fish showed signs of tenacibaculosis which has been observed during other jellyfish related mortality events [6,32] and which has been known to cause large mortality events in Atlantic salmon without the presence of jellyfish (personal observations). Also, tenacibaculosis related mortalities at both sites seemed to have started before the jellyfish bloom. The difference in the presentation of the jellyfish damage in this case, when compared to other cases in the literature could be due to this being a *D*. *typicum* bloom and its potential for causing damage to fish has not been studied.

The histological, SEM and TEM investigations into the presence of bacteria on the jellyfish revealed that the thread-like structures observed in the mouth region in our samples were cilia and not bacteria (*Tenacibaculum* spp.), in contrast with what is concluded by Delannoy, Houghton [21] and Ferguson, Christian [10]. The lack of bacteria on the surface of the jellyfish, including the mouth region could be due to the preparation of the samples; however, the authors do not think so due to the fact that identical results were found with histology, SEM and TEM, and that bacteria were observed on similarly prepared samples of fish tissues. A recent study by Viver, Orellana [56] revealed *Tenacibaculum*-like cells in the gastric filaments of the jellyfish *Cotylorhiza tuberculata*. This indicates that the bacteria might be present internally and not on the surface of the jellyfish.

The nematocysts present in some of the examined fish tissue and the associated pathological changes match what is known about their mechanism of action [18]. The authors observed embedded nematocysts with *Tenacibaculum*-like bacteria close to or attached to them (Fig 11B), as well as holes likely to be a result of nematocysts being ripped out. It is natural to assume that the holes provide an entry point for pathogens; in this case *Tenacibaculum*, which seems to have a high affinity for the underlying collagen rich dermis layer [31]. There is evidence of this in the histology sections and the SEM sections show dividing *Tenacibaculum*-like bacteria within the collagen layer of the skin (Fig 10B).

In this study, the smolts that had been recently transferred to saltwater (five net-pens at site 1 and six net-pens at site 2) were more affected in terms of total mortality when compared to the smolts that had been in saltwater for longer. This is likely a result of smolts going through a transition phase from freshwater, which has a significant impact on the physiology and immunology of the fish, and also the skin microbiota [57]. Interestingly, it looks like ‘healthy’ smolts acquire a certain level of *Tenacibaculum* spp. in their skin microbiota after only a few weeks in saltwater [57], and these bacteria seem to be part of the ‘normal’ skin microbiota of Atlantic salmon [55,58]. The real-time RT-PCR results showed that there was a higher bacterial load between moribund and randomly sampled ‘healthy’ fish, which may indicate that a certain level of the bacteria is required to cause disease.

There is a complex relationship between bacterio-, phyto- and zooplankton and jellyfish communities in the environment, which make determining the reservoirs and vectors of pathogenic bacteria difficult. Flavobacteriaceae levels, including *Tenacibaculum*, have been shown to be associated with elevated levels of organic material (e.g. phytoplankton blooms) in the environment due to their ability to decompose complex molecules [59–61]. Phytoplankton blooms frequently occur in the Barents Sea (the sea surrounding the Northern most part of Norway) [62–65]. This was also the case in 2015 when a phytoplankton bloom occurred in close proximity to the sites approximately one month prior to the mortality events (NASA Worldview, https://goo.gl/oq8Sb3). As a result, levels of Flavobacteriaceae present around the farm sites are expected to be high during these blooms when there is a high organic load in the water [59,61,66,67]. Different clades of Flavobacteriaceae will dominate at different times of the phytoplankton bloom cycles [59,60,66,68,69], and *Tenacibaculum* spp. tend to be later in the cycle when the plankton are decomposing [70]. The timeline of these cycles match what was seen in this study with the tenacibaculosis outbreaks starting 4 to 6 weeks after the onset of the phytoplankton blooms.

Jellyfish levels also react to organic load, and blooms often occur as a result of increased levels of zooplankton which generally lags that of phytoplankton blooms by a month [3,71,72]. All known described jellyfish in Phylum Cnidaria from the Barents sea forage on zooplankton [72]; it is therefore likely to be the case for *D*. *typicum*. It is also shown that jellyfish blooms can have an effect on the bacterial community composition in the vicinity of the bloom; as was shown in a study where the presence of the jellyfish *Mnemiopsis leidy* was associated with increased levels and prevalence of Flavobacteriaceae [73].

The earliest recorded bloom of *D*. *typicum* was near the British Isles in the late 1800s and the presence of the species has been reported in much of the boreal-circumpolar region [40,41]. The presence of *D*. *typicum* in Northern Norwegian waters may not be a new finding, but with new areas being used for salmon aquaculture their presence could be a rising issue. The ability of *D*. *typicum* to rapidly propagate and cause massive blooms is due to its ability of repeated transverse fission [40].

*D*. *typicum* is described as being friable [41], which was confirmed during field sampling, and can therefore easily break up into pieces that are still capable of stinging fish [6]. This might be a concern in saltwater semi-enclosed and closed systems, which generally pump in saltwater that may contain pieces or whole jellyfish as was described by Hosteland [74]. The jellyfish may be accompanied by pathogens, in particular some members of Flavobacteriaceae that are expected to be high in times of blooms, which could further exacerbate the situation. The introduction of pathogens into a closed system has already been noted for *Tenacibaculum* [75]. This is likely to be an issue for well-boat fish transports as well.

## Conclusions

Based on the findings of this study, *D*. *typicum* is unlikely to be a vector for the fish pathogenic species *T*. *finnmarkense*. However, these jellyfish are likely to cause enough damage physically through their nematocytes to result in a route of infection for pathogenic environmental or opportunistic skin bacteria.

Smolts go through an intense transition when transferred to saltwater, linked to a shift in skin microbiota, making them more susceptible to environmental stressors; therefore, increased knowledge is needed as to when and where to transfer fish into saltwater. Possible options, such as closed post-smolt facilities using treated saltwater could be a mitigation tool for outbreaks like the ones described in this study.

## Supporting information

S1 Fig SEM micrograph of gills from a moribund fish. Secondary lamellae showing intact microridges of the epidermis. There are no pathogens or nematocysts present.
(TIF)

S2 Fig SEM micrograph of an eye from a moribund fish with "cloudy" eyes. (A) Filamentous bacteria present on the surface of the cornea. (B) Nematocyst penetrating the cornea of the eye.
(TIF)

S3 Fig SEM micrograph of a skin lesion from a moribund fish. Nematocysts can be seen embedded in the skin with holes (h) present that are likely the results of them being ripped out.
(TIF)

S1 Table Jellyfish real-time RT-PCR results. Ct values of the jellyfish real-time RT-PCR analysis.
(DOCX)

S2 Table Fish real-time RT-PCR results. Ct values of the fish real-time RT-PCR analysis.
(DOCX)
