# Correction: Concurrent jellyfish blooms and tenacibaculosis outbreaks in Northern Norwegian Atlantic salmon (*Salmo salar*) farms

**DOI:** 10.1371/journal.pone.0190762

**Published:** 2018-01-02

**Authors:** Sverre Bang Småge, Øyvind Jakobsen Brevik, Kathleen Frisch, Kuninori Watanabe, Henrik Duesund, Are Nylund

The scale bars for Fig 8 and Fig 9 are incorrect. The authors have provided corrected versions here.

**Fig 8:**
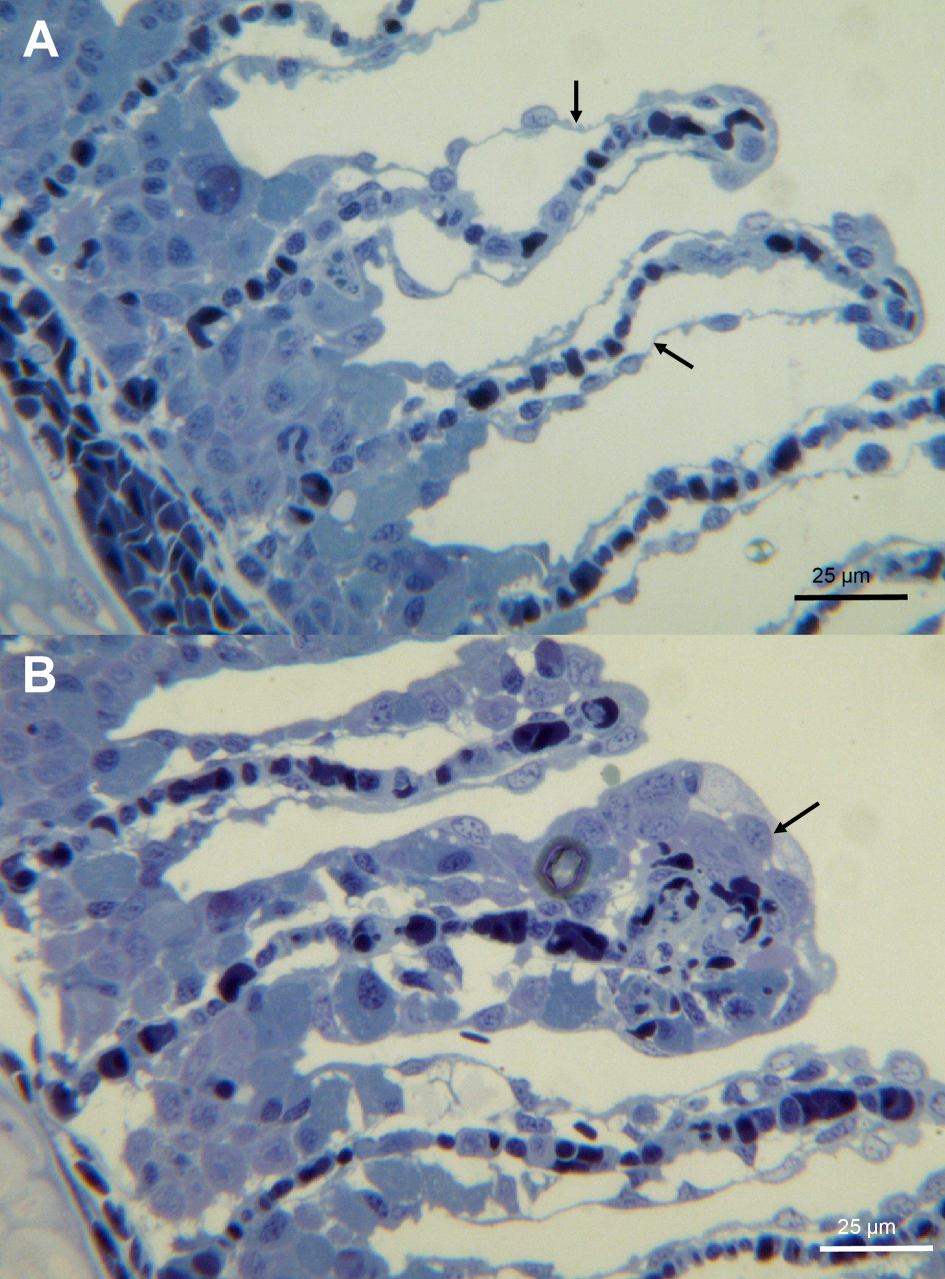
Histology of gills from moribund fish. (A) A toluidine blue stained section of gills showing epithelial lifting (arrow). (B) A toluidine blue stained section of gills showing foci of hypertrophy (arrow).

**Fig 9:**
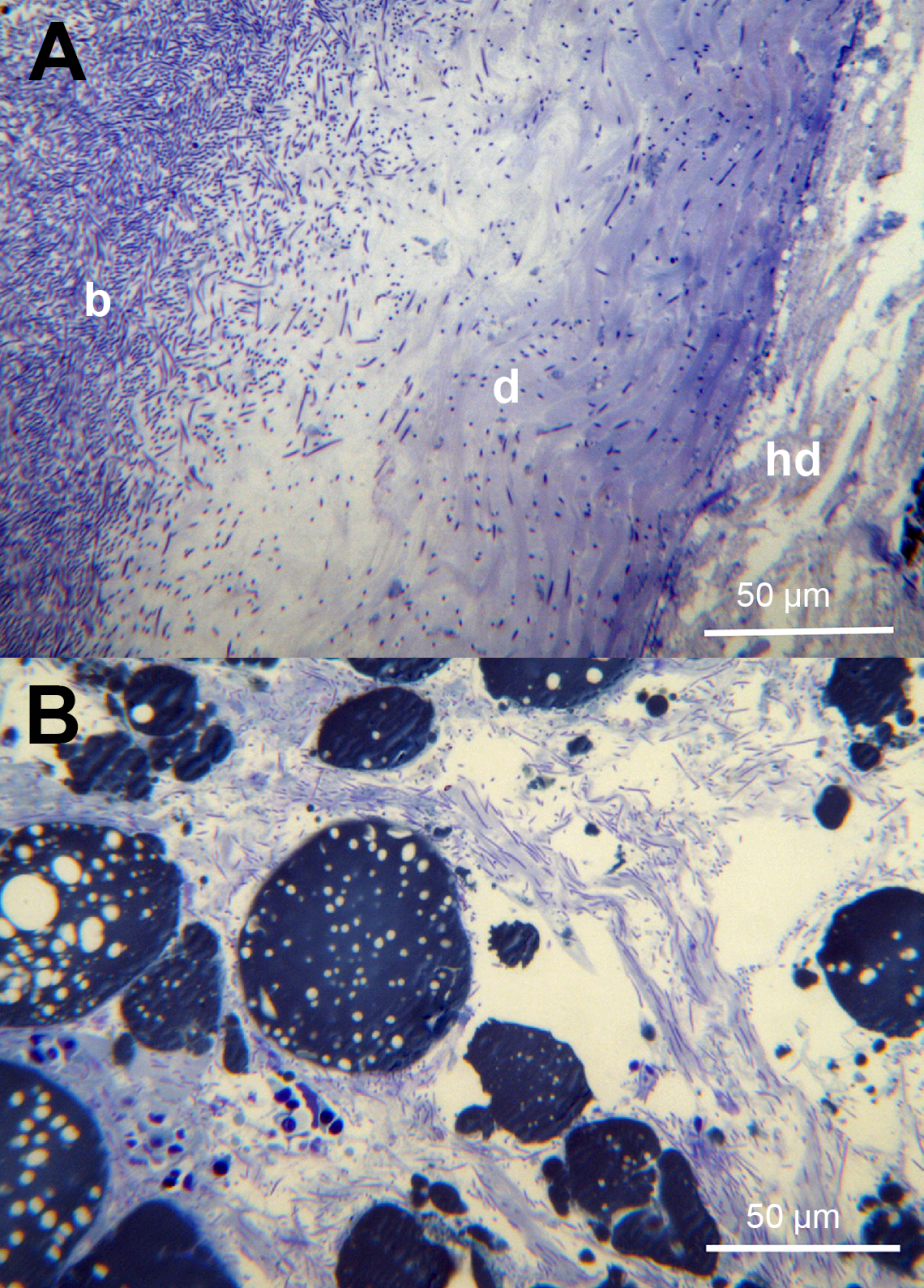
Histology of skin from moribund fish. (A) A toluidine blue stained section of skin showing complete loss of epidermis where the bacteria (b) are present and infiltration of the stratum compactum of the dermis (d). This section does not appear to have bacteria infiltrating the hypodermis (hd). (B) A toluidine blue stained section of the skin hypodermis showing large number of bacteria.
