# Supplementary figures and images for: Concurrent jellyfish blooms and tenacibaculosis outbreaks in Northern Norwegian Atlantic salmon (Salmo salar) farms
Source: PLoS One. 2017 Nov 2;12(11):e0187476. doi: 10.1371/journal.pone.0187476 (PMC5667831; doi:10.1371/journal.pone.0187476)

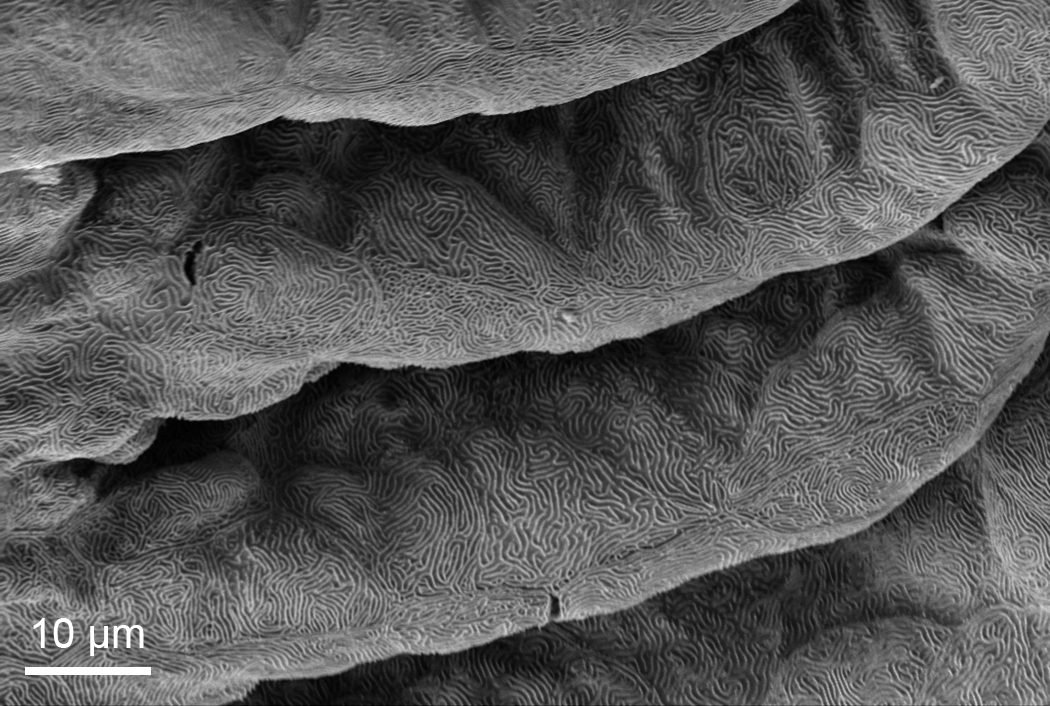

Supplement: S1 Fig — Secondary lamellae showing intact microridges of the epidermis. There are no pathogens or nematocysts present. (TIF) [file pone.0187476.s001.tif]

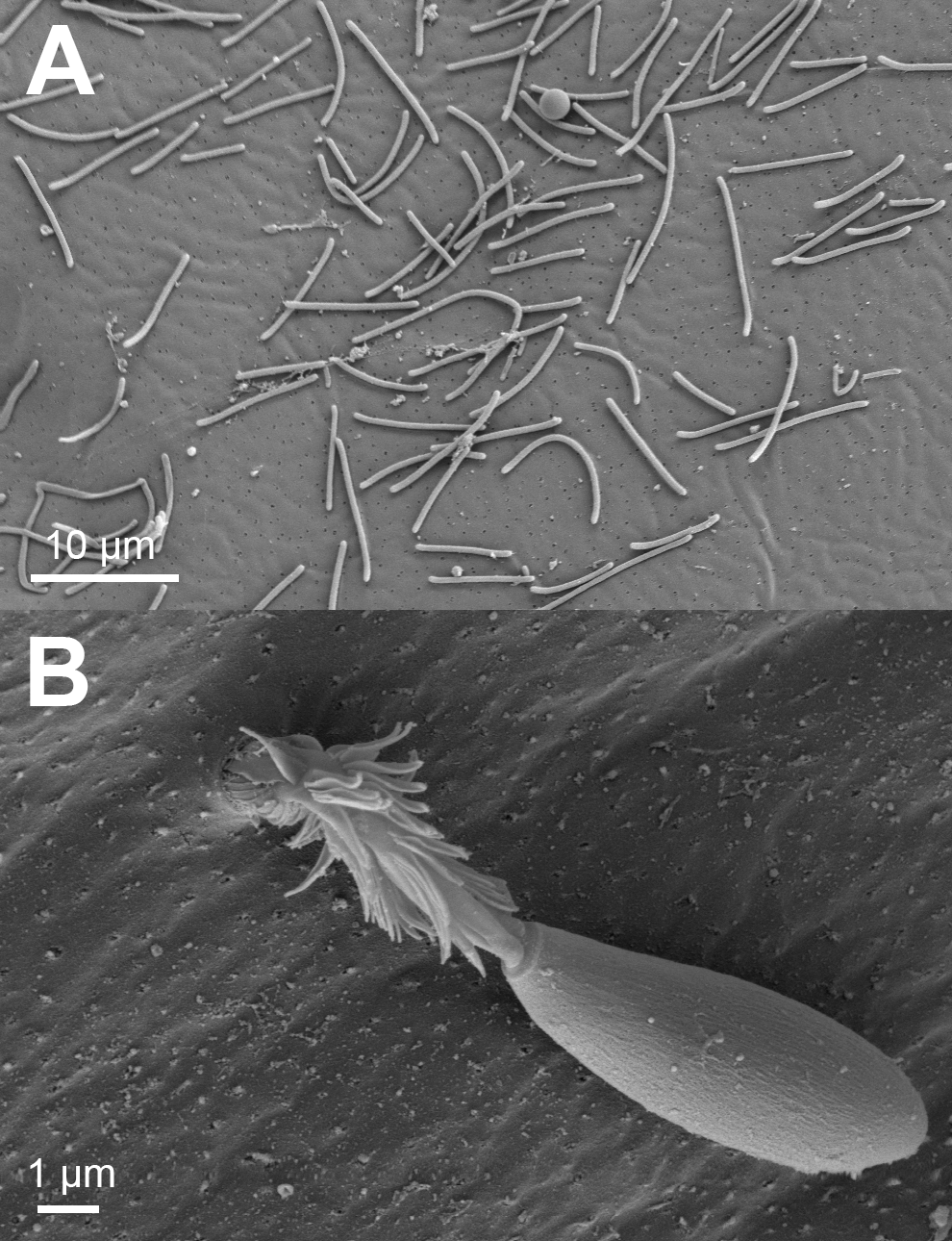

Supplement: S2 Fig — (A) Filamentous bacteria present on the surface of the cornea. (B) Nematocyst penetrating the cornea of the eye. (TIF) [file pone.0187476.s002.tif]

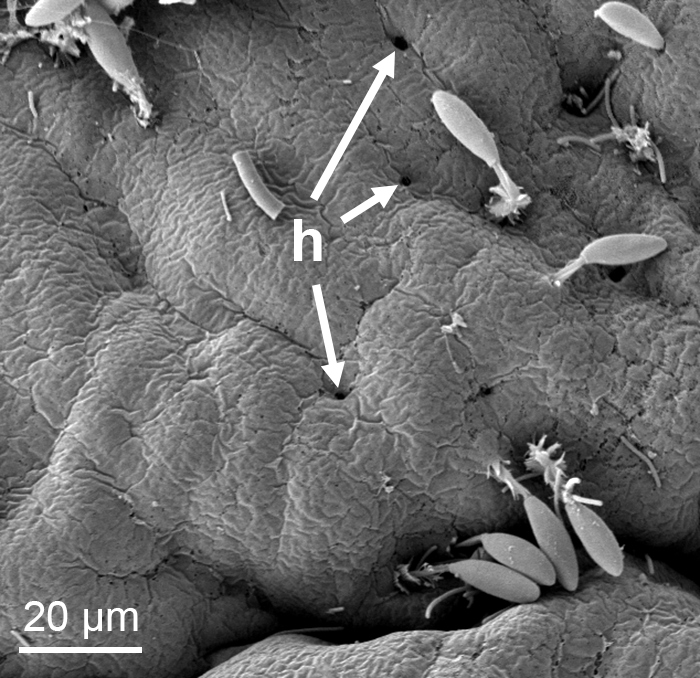

Supplement: S3 Fig — Nematocysts can be seen embedded in the skin with holes (h) present that are likely the results of them being ripped out. (TIF) [file pone.0187476.s003.tif]
