# Supplementary material for: Concurrent jellyfish blooms and tenacibaculosis outbreaks in Northern Norwegian Atlantic salmon (Salmo salar) farms
Source: PLoS One. 2017 Nov 2;12(11):e0187476. doi: 10.1371/journal.pone.0187476 (PMC5667831; doi:10.1371/journal.pone.0187476)
Supplement: S1 Table — Ct values of the jellyfish real-time RT-PCR analysis. (DOCX) [file pone.0187476.s004.docx]

| **Sample Point** | **Sample Name** | **SAL Ct-value** | **Tb tuf Ct-value** |
| --- | --- | --- | --- |
| S1E1 | 2a1 | 23.19 | Undetermined |
| S1E1 | 2a10 | 23.08 | 38.15 |
| S1E1 | 2a11 | 23.75 | Undetermined |
| S1E1 | 2a12 | 23.72 | Undetermined |
| S1E1 | 2a13 | 23.80 | Undetermined |
| S1E1 | 2a14 | 23.72 | Undetermined |
| S1E1 | 2a15 | 23.56 | Undetermined |
| S1E1 | 2a16 | 23.27 | Undetermined |
| S1E1 | 2a17 | 24.71 | Undetermined |
| S1E1 | 2a18 | 22.98 | Undetermined |
| S1E1 | 2a19 | 22.97 | Undetermined |
| S1E1 | 2a2 | 24.15 | Undetermined |
| S1E1 | 2a20 | 22.20 | 36.62 |
| S1E1 | 2a23 | N/A | Undetermined |
| S1E1 | 2a24 | N/A | Undetermined |
| S1E1 | 2a25 | N/A | Undetermined |
| S1E1 | 2a26 | N/A | Undetermined |
| S1E1 | 2a3 | 24.29 | Undetermined |
| S1E1 | 2a4 | 23.66 | Undetermined |
| S1E1 | 2a5 | 24.01 | Undetermined |
| S1E1 | 2a6 | 24.54 | Undetermined |
| S1E1 | 2a7 | 23.79 | Undetermined |
| S1E1 | 2a8 | 24.00 | Undetermined |
| S1E1 | 2a9 | 24.09 | Undetermined |
| S1 | a1 | 24.17 | Undetermined |
| S2 | a1 | 24.94 | 35.27 |
| S1 | a10 | N/A | Undetermined |
| S2 | a10 | 24.89 | Undetermined |
| S1 | a11 | N/A | Undetermined |
| S2 | a11 | 24.84 | Undetermined |
| S1 | a12 | N/A | 38.02 |
| S2 | a12 | 24.72 | 36.24 |
| S1 | a13 | N/A | Undetermined |
| S2 | a13 | 24.38 | Undetermined |
| S1 | a14 | N/A | Undetermined |
| S2 | a14 | 25.00 | Undetermined |
| S1 | a15 | N/A | Undetermined |
| S2 | a15 | 25.01 | Undetermined |
| S1 | a16 | N/A | Undetermined |
| S2 | a16 | 24.52 | 36.34 |
| S1 | a18 | N/A | Undetermined |
| S2 | a18 | 24.75 | Undetermined |
| S1 | a19 | N/A | Undetermined |
| S2 | a19 | 25.28 | Undetermined |
| S1 | a2 | 24.19 | Undetermined |
| S2 | a2 | 24.73 | Undetermined |
| S1 | a20 | N/A | 36.62 |
| S2 | a20 | 24.28 | Undetermined |
| S1 | a22 | N/A | Undetermined |
| S2 | a22 | 25.28 | Undetermined |
| S2 | a23 | 25.92 | Undetermined |
| S2 | a24 | 25.27 | Undetermined |
| S2 | a25 | 25.95 | Undetermined |
| S2 | a26 | 25.52 | Undetermined |
| S2 | a27 | 25.37 | Undetermined |
| S2 | a28 | 25.68 | Undetermined |
| S2 | a29 | 25.51 | Undetermined |
| S1 | a3 | 24.00 | Undetermined |
| S2 | a30 | 25.48 | Undetermined |
| S1 | a4 | 24.84 | Undetermined |
| S2 | a4 | 24.84 | Undetermined |
| S1 | a5 | 24.60 | Undetermined |
| S2 | a5 | 25.57 | Undetermined |
| S1 | a6 | 24.75 | Undetermined |
| S2 | a6 | 24.11 | 36.36 |
| S1 | a7 | 23.25 | Undetermined |
| S2 | a7 | 24.58 | Undetermined |
| S1 | a8 | 23.09 | Undetermined |
| S2 | a8 | 24.66 | Undetermined |
| S1 | a9 | 23.49 | Undetermined |
| S2 | a9 | 24.44 | Undetermined |
| S2E1 | b1 | 25.42 | Undetermined |
| S2E1 | b10 | 25.57 | Undetermined |
| S2E1 | b11 | 24.73 | Undetermined |
| S2E1 | b12 | 25.02 | Undetermined |
| S2E1 | b13 | 25.05 | Undetermined |
| S2E1 | b14 | 24.48 | Undetermined |
| S2E1 | b15 | 24.01 | Undetermined |
| S2E1 | b16 | 24.19 | Undetermined |
| S2E1 | b17 | 24.84 | Undetermined |
| S2E1 | b18 | 24.66 | 36.55 |
| S2E1 | b19 | 24.49 | Undetermined |
| S2E1 | b2 | 25.02 | Undetermined |
| S2E1 | b20 | 24.17 | Undetermined |
| S2E1 | b21 | 24.05 | Undetermined |
| S2E1 | b22 | 23.59 | Undetermined |
| S2E1 | b23 | 24.59 | Undetermined |
| S2E1 | b24 | 24.83 | Undetermined |
| S2E1 | b25 | 24.51 | Undetermined |
| S2E1 | b3 | 25.74 | Undetermined |
| S2E1 | b4 | 25.57 | Undetermined |
| S2E1 | b5 | 25.28 | Undetermined |
| S2E1 | b6 | 26.20 | Undetermined |
| S2E1 | b7 | 25.04 | Undetermined |
| S2E1 | b8 | 25.75 | Undetermined |
| S2E1 | b9 | 24.99 | Undetermined |
| S2E2 | c1 | 24.12 | Undetermined |
| S2E2 | c2 | 23.25 | Undetermined |
| S2E2 | c3 | 24.66 | Undetermined |
| S2E2 | c4 | 23.77 | Undetermined |
| S2E2 | c5 | 24.27 | Undetermined |
| S2E2 | c6 | 23.21 | Undetermined |
| S2E3 | d1 | 24.49 | Undetermined |
| S2E3 | d2 | 23.82 | Undetermined |
| S2E3 | d3 | 23.63 | Undetermined |
| S2E3 | d4 | 23.92 | Undetermined |
| S2E3 | d5 | 24.01 | 35.51 |
| S2E3 | d6 | 22.92 | Undetermined |
| S2E3 | d7 | 23.88 | Undetermined |
| S2E3 | d8 | Undetermined | Undetermined |
