# Supplementary material for: Concurrent jellyfish blooms and tenacibaculosis outbreaks in Northern Norwegian Atlantic salmon (Salmo salar) farms
Source: PLoS One. 2017 Nov 2;12(11):e0187476. doi: 10.1371/journal.pone.0187476 (PMC5667831; doi:10.1371/journal.pone.0187476)
Supplement: S2 Table — Ct values of the fish real-time RT-PCR analysis. (DOCX) [file pone.0187476.s005.docx]

| **Farm Site** | **Fish Number** | **Group** | **Status** | **Generation** | **Weight (g)** | **Length (cm)** | **ELF1A Ct-value** | **Tb tuf Ct-value** | **Mv ompA Ct-value** |
| --- | --- | --- | --- | --- | --- | --- | --- | --- | --- |
| S1 | M10-7 | Pen 10 | mortality | autumn | N/A | N/A | 15.31 | 12.83 | Undetermined |
| S1 | M10-6 | Pen 10 | mortality | autumn | N/A | N/A | 16.13 | 14.09 | Undetermined |
| S1 | M10-10 | Pen 10 | mortality | autumn | N/A | N/A | 15.61 | 15.86 | Undetermined |
| S1 | M10-5 | Pen 10 | mortality | autumn | N/A | N/A | 17.33 | 16.31 | Undetermined |
| S1 | M10-4 | Pen 10 | mortality | autumn | N/A | N/A | 15.85 | 16.35 | Undetermined |
| S1 | M10-1 | Pen 10 | mortality | autumn | N/A | N/A | 16.90 | 16.97 | Undetermined |
| S1 | M10-2 | Pen 10 | mortality | autumn | N/A | N/A | 15.17 | 17.29 | Undetermined |
| S1 | M10-8 | Pen 10 | mortality | autumn | N/A | N/A | 16.50 | 17.97 | Undetermined |
| S1 | M10-3 | Pen 10 | mortality | autumn | N/A | N/A | 18.69 | 19.01 | Undetermined |
| S1 | M10-9 | Pen 10 | mortality | autumn | N/A | N/A | 16.83 | 24.20 | Undetermined |
| S1 | M7-7 | Pen 7 | mortality | autumn | N/A | N/A | 15.31 | 12.83 | Undetermined |
| S1 | M7-6 | Pen 7 | mortality | autumn | N/A | N/A | 16.13 | 14.09 | Undetermined |
| S1 | M7-10 | Pen 7 | mortality | autumn | N/A | N/A | 15.61 | 15.86 | Undetermined |
| S1 | M7-5 | Pen 7 | mortality | autumn | N/A | N/A | 17.33 | 16.31 | Undetermined |
| S1 | M7-4 | Pen 7 | mortality | autumn | N/A | N/A | 15.85 | 16.35 | Undetermined |
| S1 | M7-1 | Pen 7 | mortality | autumn | N/A | N/A | 16.90 | 16.97 | Undetermined |
| S1 | M7-2 | Pen 7 | mortality | autumn | N/A | N/A | 15.17 | 17.29 | Undetermined |
| S1 | M7-8 | Pen 7 | mortality | autumn | N/A | N/A | 16.50 | 17.97 | Undetermined |
| S1 | M7-3 | Pen 7 | mortality | autumn | N/A | N/A | 18.69 | 19.01 | Undetermined |
| S1 | M7-9 | Pen 7 | mortality | autumn | N/A | N/A | 16.83 | 24.20 | Undetermined |
| S1 | M8-1 | Pen 8 | mortality | autumn | N/A | N/A | 16.99 | 14.43 | Undetermined |
| S1 | M8-10 | Pen 8 | mortality | autumn | N/A | N/A | 17.25 | 15.46 | Undetermined |
| S1 | M8-5 | Pen 8 | mortality | autumn | N/A | N/A | 17.00 | 16.40 | Undetermined |
| S1 | M8-7 | Pen 8 | mortality | autumn | N/A | N/A | 16.81 | 17.15 | Undetermined |
| S1 | M8-6 | Pen 8 | mortality | autumn | N/A | N/A | 17.92 | 17.27 | Undetermined |
| S1 | M8-8 | Pen 8 | mortality | autumn | N/A | N/A | 16.65 | 17.40 | Undetermined |
| S1 | M8-4 | Pen 8 | mortality | autumn | N/A | N/A | 18.15 | 17.86 | Undetermined |
| S1 | M8-9 | Pen 8 | mortality | autumn | N/A | N/A | 16.89 | 18.56 | Undetermined |
| S1 | M8-2 | Pen 8 | mortality | autumn | N/A | N/A | 18.24 | 19.18 | Undetermined |
| S1 | M8-3 | Pen 8 | mortality | autumn | N/A | N/A | 18.31 | 23.72 | Undetermined |
| S1 | M9-2 | Pen 9 | mortality | autumn | N/A | N/A | 17.13 | 13.32 | Undetermined |
| S1 | M9-9 | Pen 9 | mortality | autumn | N/A | N/A | 16.75 | 14.67 | Undetermined |
| S1 | M9-3 | Pen 9 | mortality | autumn | N/A | N/A | 16.77 | 14.92 | Undetermined |
| S1 | M9-1 | Pen 9 | mortality | autumn | N/A | N/A | 17.37 | 15.00 | Undetermined |
| S1 | M9-5 | Pen 9 | mortality | autumn | N/A | N/A | 16.59 | 15.04 | Undetermined |
| S1 | M9-8 | Pen 9 | mortality | autumn | N/A | N/A | 17.04 | 15.14 | Undetermined |
| S1 | M9-7 | Pen 9 | mortality | autumn | N/A | N/A | 17.40 | 17.03 | Undetermined |
| S1 | M9-4 | Pen 9 | mortality | autumn | N/A | N/A | 17.66 | 17.81 | Undetermined |
| S1 | M9-6 | Pen 9 | mortality | autumn | N/A | N/A | 17.76 | 18.01 | Undetermined |
| S1 | M9-10 | Pen 9 | mortality | autumn | N/A | N/A | 17.49 | 19.33 | Undetermined |
| S2 | M1-2 | Pen 1 | random | spring | 120 | 21 | 16.66 | Undetermined | Undetermined |
| S2 | M1-13 | Pen 1 | random | spring | 174 | 23 | 17.31 | Undetermined | Undetermined |
| S2 | M1-24 | Pen 1 | random | spring | 160 | 22 | 17.35 | 35.43 | Undetermined |
| S2 | M1-3 | Pen 1 | random | spring | 139 | 23 | 17.69 | 36.00 | Undetermined |
| S2 | M1-15 | Pen 1 | random | spring | 183 | 24 | 17.71 | 35.98 | Undetermined |
| S2 | M1-20 | Pen 1 | random | spring | 100 | 20 | 17.76 | Undetermined | Undetermined |
| S2 | M1-12 | Pen 1 | random | spring | 107 | 20 | 18.46 | 32.78 | Undetermined |
| S2 | M1-19 | Pen 1 | random | spring | 145 | 22 | 18.46 | Undetermined | Undetermined |
| S2 | M1-9 | Pen 1 | random | spring | 196 | 25 | 18.46 | 33.63 | Undetermined |
| S2 | M1-10 | Pen 1 | random | spring | 113 | 21 | 18.48 | 34.58 | Undetermined |
| S2 | M1-11 | Pen 1 | random | spring | 128 | 21 | 18.63 | 36.05 | Undetermined |
| S2 | M1-29 | Pen 1 | random | spring | 165 | 21 | 18.79 | Undetermined | Undetermined |
| S2 | M1-22 | Pen 1 | random | spring | 132 | 21 | 18.93 | Undetermined | Undetermined |
| S2 | M1-6 | Pen 1 | random | spring | 138 | 22 | 18.93 | Undetermined | Undetermined |
| S2 | M1-30 | Pen 1 | random | spring | 240 | 27 | 18.96 | 36.42 | Undetermined |
| S2 | M1-17 | Pen 1 | random | spring | 337 | 30 | 19.03 | Undetermined | Undetermined |
| S2 | M1-23 | Pen 1 | random | spring | 172 | 23 | 19.06 | Undetermined | Undetermined |
| S2 | M1-1 | Pen 1 | random | spring | 130 | 23 | 19.12 | 29.23 | Undetermined |
| S2 | M1-21 | Pen 1 | random | spring |  |  | 19.21 | Undetermined | Undetermined |
| S2 | M1-18 | Pen 1 | random | spring | 121 | 22 | 19.44 | Undetermined | Undetermined |
| S2 | M1-4 | Pen 1 | random | spring | 227 | 26 | 19.47 | 36.31 | Undetermined |
| S2 | M1-16 | Pen 1 | random | spring | 202 | 25 | 19.71 | Undetermined | Undetermined |
| S2 | M1-5 | Pen 1 | random | spring | 145 | 23 | 19.85 | 34.25 | Undetermined |
| S2 | M1-8 | Pen 1 | random | spring | 73 | 18 | 20.27 | 36.29 | Undetermined |
| S2 | M1-14 | Pen 1 | random | spring | 176 | 22 | 20.96 | 35.74 | Undetermined |
| S2 | M1-27 | Pen 1 | random | spring | 143 | 23 | 20.99 | Undetermined | Undetermined |
| S2 | M1-7 | Pen 1 | random | spring | 132 | 21 | 21.52 | 36.59 | Undetermined |
| S2 | M1-25 | Pen 1 | random | spring | 165 | 23 | 21.54 | 36.89 | Undetermined |
| S2 | M1-26 | Pen 1 | random | spring | 127 | 20 | 21.74 | Undetermined | Undetermined |
| S2 | M1-28 | Pen 1 | random | spring | 180 | 24 | 22.27 | Undetermined | Undetermined |
| S2 | M1-33 | Pen 1 | mortality | spring | 172 | 22 | 21.92 | 31.99 | Undetermined |
| S2 | M1-32 | Pen 1 | mortality | spring | 145 | 22 | 20.73 | 29.54 | Undetermined |
| S2 | M1-34 | Pen 1 | mortality | spring | 47 | 17 | 19.21 | 21.23 | Undetermined |
| S2 | M1-35 | Pen 1 | mortality | spring | 109 | 21 | 26.94 | 19.89 | Undetermined |
| S2 | M1-31 | Pen 1 | mortality | spring | 84 | 20 | 20.43 | 15.99 | Undetermined |
| S2 | M10-28 | Pen 10 | random | spring | 128 | 22 | 19.18 | Undetermined | Undetermined |
| S2 | M10-26 | Pen 10 | random | spring | 150 | 25 | 19.98 | 34.32 | Undetermined |
| S2 | M10-7 | Pen 10 | random | spring | 193 | 26 | 19.99 | Undetermined | Undetermined |
| S2 | M10-30 | Pen 10 | random | spring | 161 | 23 | 20.47 | 36.38 | Undetermined |
| S2 | M10-8 | Pen 10 | random | spring | 163 | 26 | 20.65 | 34.17 | Undetermined |
| S2 | M10-12 | Pen 10 | random | spring | 125 | 22 | 20.87 | Undetermined | Undetermined |
| S2 | M10-9 | Pen 10 | random | spring | 153 | 23 | 20.94 | 36.51 | Undetermined |
| S2 | M10-17 | Pen 10 | random | spring | 137 | 22 | 20.99 | 36.35 | Undetermined |
| S2 | M10-29 | Pen 10 | random | spring | 247 | 35 | 21.27 | Undetermined | Undetermined |
| S2 | M10-14 | Pen 10 | random | spring | 95 | 21 | 21.33 | Undetermined | Undetermined |
| S2 | M10-3 | Pen 10 | random | spring | 130 | 22 | 21.39 | 36.36 | Undetermined |
| S2 | M10-10 | Pen 10 | random | spring | 109 | 24 | 21.50 | Undetermined | Undetermined |
| S2 | M10-19 | Pen 10 | random | spring | 114 | 22 | 21.67 | Undetermined | Undetermined |
| S2 | M10-2 | Pen 10 | random | spring | 110 | 22 | 21.84 | 33.20 | Undetermined |
| S2 | M10-20 | Pen 10 | random | spring | 159 | 23 | 22.05 | Undetermined | Undetermined |
| S2 | M10-21 | Pen 10 | random | spring | 112 | 25 | 22.10 | 34.77 | Undetermined |
| S2 | M10-11 | Pen 10 | random | spring | 113 | 21 | 22.12 | 34.73 | Undetermined |
| S2 | M10-15 | Pen 10 | random | spring | 164 | 24 | 22.36 | Undetermined | Undetermined |
| S2 | M10-22 | Pen 10 | random | spring | 113 | 22 | 22.41 | Undetermined | Undetermined |
| S2 | M10-18 | Pen 10 | random | spring | 128 | 23 | 22.76 | 36.26 | Undetermined |
| S2 | M10-27 | Pen 10 | random | spring | 206 | 26 | 22.80 | Undetermined | Undetermined |
| S2 | M10-4 | Pen 10 | random | spring | 102 | 22 | 22.82 | Undetermined | Undetermined |
| S2 | M10-16 | Pen 10 | random | spring | 134 | 23 | 22.85 | Undetermined | Undetermined |
| S2 | M10-23 | Pen 10 | random | spring | 114 | 22 | 22.95 | Undetermined | Undetermined |
| S2 | M10-24 | Pen 10 | random | spring | 155 | 24 | 23.10 | Undetermined | Undetermined |
| S2 | M10-5 | Pen 10 | random | spring | 182 | 25 | 23.25 | Undetermined | Undetermined |
| S2 | M10-13 | Pen 10 | random | spring | 126 | 21 | 23.35 | 36.33 | Undetermined |
| S2 | M10-25 | Pen 10 | random | spring | 137 | 23 | 23.46 | 35.24 | Undetermined |
| S2 | M10-35 | Pen 10 | mortality | spring | 137 | 23 | 20.69 | 31.72 | Undetermined |
| S2 | M10-6 | Pen 10 | random | spring | 91 | 23 | 23.86 | 35.21 | Undetermined |
| S2 | M10-1 | Pen 10 | random | spring | 88 | 21 | 24.62 | 29.14 | Undetermined |
| S2 | M10-32 | Pen 10 | mortality | spring | 92 | 22 | 21.55 | 26.50 | Undetermined |
| S2 | M10-34 | Pen 10 | mortality | spring | 128 | 24 | 21.19 | 24.25 | Undetermined |
| S2 | M10-31 | Pen 10 | mortality | spring | 95 | 23 | 20.49 | 21.71 | Undetermined |
| S2 | M10-33 | Pen 10 | mortality | spring | 82 | 22 | 23.15 | 18.36 | Undetermined |
| S2 | M11-12 | Pen 11 | random | spring | 181 | 23 | 16.97 | Undetermined | Undetermined |
| S2 | M11-8 | Pen 11 | random | spring | 218 | 26 | 17.47 | 34.37 | Undetermined |
| S2 | M11-16 | Pen 11 | random | spring | 166 | 24 | 17.80 | 35.77 | Undetermined |
| S2 | M11-7 | Pen 11 | random | spring | 137 | 24 | 17.91 | Undetermined | Undetermined |
| S2 | M11-17 | Pen 11 | random | spring | 177 | 24 | 17.93 | Undetermined | Undetermined |
| S2 | M11-11 | Pen 11 | random | spring | 192 | 25 | 18.28 | Undetermined | Undetermined |
| S2 | M11-9 | Pen 11 | random | spring | 205 | 27 | 18.38 | 33.31 | Undetermined |
| S2 | M11-15 | Pen 11 | random | spring | 147 | 22 | 18.56 | Undetermined | Undetermined |
| S2 | M11-13 | Pen 11 | random | spring | 95 | 20 | 18.60 | 33.73 | Undetermined |
| S2 | M11-1 | Pen 11 | random | spring | 106 | 25 | 18.69 | 30.81 | Undetermined |
| S2 | M11-20 | Pen 11 | random | spring | 141 | 23 | 18.78 | 33.98 | Undetermined |
| S2 | M11-10 | Pen 11 | random | spring | 177 | 25 | 18.81 | 35.18 | Undetermined |
| S2 | M11-21 | Pen 11 | random | spring | 195 | 25 | 18.95 | Undetermined | Undetermined |
| S2 | M11-22 | Pen 11 | random | spring | 147 | 22 | 19.23 | Undetermined | Undetermined |
| S2 | M11-14 | Pen 11 | random | spring | 214 | 25 | 19.26 | Undetermined | Undetermined |
| S2 | M11-29 | Pen 11 | random | spring | 159 | 24 | 19.38 | 36.30 | Undetermined |
| S2 | M11-2 | Pen 11 | random | spring | 106 | 25 | 19.71 | 36.17 | Undetermined |
| S2 | M11-18 | Pen 11 | random | spring | 178 | 23 | 19.94 | 36.32 | Undetermined |
| S2 | M11-5 | Pen 11 | random | spring | 159 | 24 | 20.27 | 34.21 | Undetermined |
| S2 | M11-27 | Pen 11 | random | spring | 188 | 25 | 20.67 | Undetermined | Undetermined |
| S2 | M11-3 | Pen 11 | random | spring | 120 | 21 | 20.69 | Undetermined | Undetermined |
| S2 | M11-24 | Pen 11 | random | spring | 200 | 25 | 20.80 | 35.05 | Undetermined |
| S2 | M11-6 | Pen 11 | random | spring | 217 | 28 | 20.89 | 36.58 | Undetermined |
| S2 | M11-19 | Pen 11 | random | spring | 118 | 21 | 20.99 | Undetermined | Undetermined |
| S2 | M11-25 | Pen 11 | random | spring | 146 | 25 | 21.02 | Undetermined | Undetermined |
| S2 | M11-23 | Pen 11 | random | spring | 98 | 21 | 21.10 | Undetermined | Undetermined |
| S2 | M11-26 | Pen 11 | random | spring | 170 | 24 | 21.13 | Undetermined | Undetermined |
| S2 | M11-4 | Pen 11 | random | spring | 240 | 26 | 21.31 | Undetermined | Undetermined |
| S2 | M11-28 | Pen 11 | random | spring | 174 | 24 | 21.69 | Undetermined | Undetermined |
| S2 | M11-35 | Pen 11 | mortality | spring | 90 | 22 | 18.99 | 32.12 | Undetermined |
| S2 | M11-32 | Pen 11 | mortality | spring | 81 | 19 | 21.83 | 28.51 | 35.99 |
| S2 | M11-30 | Pen 11 | random | spring | 157 | 23 | 25.57 | Undetermined | Undetermined |
| S2 | M11-34 | Pen 11 | mortality | spring | 152 | 25 | 20.69 | 24.45 | 32.57 |
| S2 | M11-33 | Pen 11 | mortality | spring | 117 | 23 | 21.89 | 22.55 | Undetermined |
| S2 | M11-31 | Pen 11 | mortality | spring | 125 | 21 | 22.51 | 18.38 | Undetermined |
| S2 | M12-5 | Pen 12 | random | spring | 122 | 21 | 18.04 | 35.39 | Undetermined |
| S2 | M12-12 | Pen 12 | random | spring | 208 | 26 | 19.18 | Undetermined | Undetermined |
| S2 | M12-21 | Pen 12 | random | spring | 247 | 25 | 19.44 | Undetermined | Undetermined |
| S2 | M12-18 | Pen 12 | random | spring | 245 | 27 | 19.77 | Undetermined | Undetermined |
| S2 | M12-14 | Pen 12 | random | spring | 239 | 27 | 20.16 | Undetermined | Undetermined |
| S2 | M12-13 | Pen 12 | random | spring | 197 | 24 | 20.26 | Undetermined | Undetermined |
| S2 | M12-6 | Pen 12 | random | spring | 118 | 22 | 20.29 | 36.44 | Undetermined |
| S2 | M12-4 | Pen 12 | random | spring | 132 | 21 | 20.39 | Undetermined | Undetermined |
| S2 | M12-10 | Pen 12 | random | spring | 156 | 24 | 20.77 | Undetermined | Undetermined |
| S2 | M12-3 | Pen 12 | random | spring | 192 | 26 | 20.95 | Undetermined | Undetermined |
| S2 | M12-11 | Pen 12 | random | spring | 202 | 26 | 21.20 | Undetermined | Undetermined |
| S2 | M12-1 | Pen 12 | random | spring | 256 | 27 | 21.74 | Undetermined | Undetermined |
| S2 | M12-25 | Pen 12 | random | spring | 125 | 21 | 21.95 | Undetermined | Undetermined |
| S2 | M12-23 | Pen 12 | random | spring | 114 | 21 | 22.14 | Undetermined | Undetermined |
| S2 | M12-7 | Pen 12 | random | spring | 237 | 27 | 22.28 | Undetermined | Undetermined |
| S2 | M12-33 | Pen 12 | mortality | spring | 70 | 18 | 26.69 | 33.98 | Undetermined |
| S2 | M12-19 | Pen 12 | random | spring | 237 | 26 | 22.46 | Undetermined | Undetermined |
| S2 | M12-27 | Pen 12 | random | spring | 91 | 19 | 23.19 | Undetermined | Undetermined |
| S2 | M12-24 | Pen 12 | random | spring | 136 | 21 | 23.30 | Undetermined | Undetermined |
| S2 | M12-15 | Pen 12 | random | spring | 69 | 19 | 23.35 | Undetermined | Undetermined |
| S2 | M12-26 | Pen 12 | random | spring | 162 | 22 | 23.88 | Undetermined | Undetermined |
| S2 | M12-30 | Pen 12 | random | spring | 126 | 21 | 24.25 | Undetermined | Undetermined |
| S2 | M12-31 | Pen 12 | mortality | spring | 128 | 21 | 25.95 | 30.15 | Undetermined |
| S2 | M12-22 | Pen 12 | random | spring | 112 | 20 | 24.41 | Undetermined | Undetermined |
| S2 | M12-8 | Pen 12 | random | spring | 181 | 24 | 24.49 | Undetermined | Undetermined |
| S2 | M12-29 | Pen 12 | random | spring | 144 | 22 | 24.73 | 32.75 | Undetermined |
| S2 | M12-16 | Pen 12 | random | spring | 147 | 23 | 24.88 | Undetermined | Undetermined |
| S2 | M12-20 | Pen 12 | random | spring | 92 | 20 | 24.89 | Undetermined | Undetermined |
| S2 | M12-9 | Pen 12 | random | spring | 169 | 25 | 24.98 | Undetermined | Undetermined |
| S2 | M12-2 | Pen 12 | random | spring | 132 | 23 | 25.05 | Undetermined | Undetermined |
| S2 | M12-28 | Pen 12 | random | spring | 123 | 21 | 25.39 | Undetermined | Undetermined |
| S2 | M12-17 | Pen 12 | random | spring | 117 | 21 | 26.13 | Undetermined | Undetermined |
| S2 | M12-32 | Pen 12 | mortality | spring | 115 | 21 | 20.76 | 24.20 | Undetermined |
| S2 | M2-4 | Pen 2 | random | spring | 165 | 23 | 18.53 | 30.10 | Undetermined |
| S2 | M2-18 | Pen 2 | random | spring | 136 | 21 | 18.55 | 31.80 | Undetermined |
| S2 | M2-16 | Pen 2 | random | spring | 172 | 24 | 18.64 | 34.31 | Undetermined |
| S2 | M2-13 | Pen 2 | random | spring | 68 | 19 | 18.80 | 34.13 | Undetermined |
| S2 | M2-19 | Pen 2 | random | spring | 214 | 24 | 18.98 | Undetermined | Undetermined |
| S2 | M2-15 | Pen 2 | random | spring | 107 | 20 | 19.09 | Undetermined | Undetermined |
| S2 | M2-17 | Pen 2 | random | spring | 135 | 20 | 19.15 | Undetermined | Undetermined |
| S2 | M2-21 | Pen 2 | random | spring | 119 | 22 | 19.44 | Undetermined | Undetermined |
| S2 | M2-2 | Pen 2 | random | spring | 172 | 22 | 19.47 | 30.07 | Undetermined |
| S2 | M2-9 | Pen 2 | random | spring | 142 | 22 | 19.67 | 35.50 | Undetermined |
| S2 | M2-14 | Pen 2 | random | spring | 131 | 20 | 19.92 | Undetermined | Undetermined |
| S2 | M2-3 | Pen 2 | random | spring | 137 | 21 | 20.17 | 31.18 | Undetermined |
| S2 | M2-12 | Pen 2 | random | spring | 174 | 22 | 20.20 | 35.59 | Undetermined |
| S2 | M2-23 | Pen 2 | random | spring | 125 | 20 | 20.47 | Undetermined | Undetermined |
| S2 | M2-27 | Pen 2 | random | spring | 210 | 24 | 20.58 | Undetermined | Undetermined |
| S2 | M2-5 | Pen 2 | random | spring | 198 | 24 | 20.70 | 33.53 | Undetermined |
| S2 | M2-22 | Pen 2 | random | spring | 147 | 21 | 20.84 | 34.02 | Undetermined |
| S2 | M2-6 | Pen 2 | random | spring | 207 | 24 | 21.04 | 39.42 | Undetermined |
| S2 | M2-11 | Pen 2 | random | spring | 204 | 25 | 21.07 | Undetermined | Undetermined |
| S2 | M2-29 | Pen 2 | random | spring | 124 | 21 | 21.31 | 36.31 | Undetermined |
| S2 | M2-25 | Pen 2 | random | spring | 171 | 23 | 21.41 | 34.41 | Undetermined |
| S2 | M2-7 | Pen 2 | random | spring | 141 | 22 | 21.48 | 33.57 | Undetermined |
| S2 | M2-28 | Pen 2 | random | spring | 139 | 21 | 22.28 | Undetermined | Undetermined |
| S2 | M2-20 | Pen 2 | random | spring | 118 | 21 | 22.41 | Undetermined | Undetermined |
| S2 | M2-30 | Pen 2 | random | spring | 124 | 21 | 24.00 | Undetermined | Undetermined |
| S2 | M2-31 | Pen 2 | mortality | spring | 81 | 18 | 25.13 | 30.14 | Undetermined |
| S2 | M2-26 | Pen 2 | random | spring | 145 | 22 | 24.49 | Undetermined | Undetermined |
| S2 | M2-24 | Pen 2 | random | spring | 179 | 22 | 24.52 | Undetermined | Undetermined |
| S2 | M2-1 | Pen 2 | random | spring | 151 | 22 | 25.10 | 30.27 | Undetermined |
| S2 | M2-10 | Pen 2 | random | spring | 184 | 24 | 25.14 | Undetermined | Undetermined |
| S2 | M2-34 | Pen 2 | mortality | spring | 131 | 21 | 25.14 | 27.63 | Undetermined |
| S2 | M2-8 | Pen 2 | random | spring | 131 | 21 | 26.61 | Undetermined | Undetermined |
| S2 | M2-35 | Pen 2 | mortality | spring | 100 | 20 | 27.50 | 24.42 | Undetermined |
| S2 | M2-32 | Pen 2 | mortality | spring | 100 | 18 | 22.16 | 23.42 | Undetermined |
| S2 | M2-33 | Pen 2 | mortality | spring | 84 | 21 | 27.66 | 22.89 | Undetermined |
| S2 | M3-9 | Pen 3 | random | spring | 180 | 27 | 18.68 | 34.62 | Undetermined |
| S2 | M3-1 | Pen 3 | random | spring | 190 | 25 | 18.91 | Undetermined | Undetermined |
| S2 | M3-4 | Pen 3 | random | spring | 260 | 30 | 19.00 | Undetermined | Undetermined |
| S2 | M3-3 | Pen 3 | random | spring | 150 | 24 | 19.19 | Undetermined | Undetermined |
| S2 | M3-13 | Pen 3 | random | spring | 235 | 30 | 19.39 | Undetermined | Undetermined |
| S2 | M3-5 | Pen 3 | random | spring | 200 | 30 | 19.73 | 35.35 | Undetermined |
| S2 | M3-8 | Pen 3 | random | spring | 130 | 25 | 19.77 | Undetermined | Undetermined |
| S2 | M3-24 | Pen 3 | random | spring | 260 | 30 | 19.92 | 35.81 | Undetermined |
| S2 | M3-26 | Pen 3 | random | spring | 100 | 20 | 20.20 | Undetermined | Undetermined |
| S2 | M3-29 | Pen 3 | random | spring | 160 | 23 | 20.23 | Undetermined | Undetermined |
| S2 | M3-22 | Pen 3 | random | spring | 210 | 23 | 20.42 | Undetermined | Undetermined |
| S2 | M3-21 | Pen 3 | random | spring | 180 | 25 | 20.43 | Undetermined | Undetermined |
| S2 | M3-25 | Pen 3 | random | spring | 100 | 21 | 20.43 | Undetermined | Undetermined |
| S2 | M3-7 | Pen 3 | random | spring | 250 | 28 | 20.52 | 33.98 | Undetermined |
| S2 | M3-28 | Pen 3 | random | spring | 220 | 26 | 20.62 | Undetermined | Undetermined |
| S2 | M3-16 | Pen 3 | random | spring | 163 | 24 | 20.70 | 35.05 | Undetermined |
| S2 | M3-12 | Pen 3 | random | spring | 125 | 26 | 21.13 | 34.46 | Undetermined |
| S2 | M3-27 | Pen 3 | random | spring | 130 | 24 | 21.25 | Undetermined | Undetermined |
| S2 | M3-19 | Pen 3 | random | spring | 158 | 24 | 21.26 | 35.30 | Undetermined |
| S2 | M3-14 | Pen 3 | random | spring | 267 | 31 | 21.29 | 36.07 | Undetermined |
| S2 | M3-10 | Pen 3 | random | spring | 180 | 27 | 21.31 | Undetermined | Undetermined |
| S2 | M3-23 | Pen 3 | random | spring | 220 | 26 | 21.55 | Undetermined | Undetermined |
| S2 | M3-15 | Pen 3 | random | spring | 150 | 24 | 21.59 | Undetermined | Undetermined |
| S2 | M3-30 | Pen 3 | random | spring | 170 | 26 | 21.71 | Undetermined | Undetermined |
| S2 | M3-2 | Pen 3 | random | spring | 250 | 27 | 21.76 | Undetermined | Undetermined |
| S2 | M3-31 | Pen 3 | mortality | spring | 219 | 28 | 20.81 | 33.80 | Undetermined |
| S2 | M3-17 | Pen 3 | random | spring | 175 | 23 | 22.67 | 35.08 | Undetermined |
| S2 | M3-11 | Pen 3 | random | spring | 225 | 27 | 23.41 | Undetermined | Undetermined |
| S2 | M3-18 | Pen 3 | random | spring | 128 | 23 | 23.58 | Undetermined | Undetermined |
| S2 | M3-6 | Pen 3 | random | spring | 140 | 23 | 23.67 | 36.41 | Undetermined |
| S2 | M3-20 | Pen 3 | random | spring | 194 | 24 | 23.76 | Undetermined | Undetermined |
| S2 | M3-32 | Pen 3 | mortality | spring | 125 | 27 | 22.98 | 28.98 | Undetermined |
| S2 | M3-34 | Pen 3 | mortality | spring | 85 | 21 | 20.00 | 25.59 | Undetermined |
| S2 | M3-33 | Pen 3 | mortality | spring | 84 | 21 | 21.55 | 24.71 | Undetermined |
| S2 | M3-35 | Pen 3 | mortality | spring | 110 | 21 | 19.81 | 24.65 | Undetermined |
| S2 | M4-12 | Pen 4 | random | autumn | 71 | 18 | 18.10 | 29.49 | Undetermined |
| S2 | M4-14 | Pen 4 | random | autumn | 66 | 18 | 18.81 | 29.73 | Undetermined |
| S2 | M4-10 | Pen 4 | random | autumn | 49 | 16 | 19.14 | 32.04 | Undetermined |
| S2 | M4-7 | Pen 4 | random | autumn | 77 | 18 | 19.25 | 33.68 | Undetermined |
| S2 | M4-28 | Pen 4 | random | autumn | 72 | 18 | 19.40 | 29.50 | Undetermined |
| S2 | M4-9 | Pen 4 | random | autumn | 80 | 17 | 19.51 | 33.71 | Undetermined |
| S2 | M4-2 | Pen 4 | random | autumn | 77 | 19 | 20.01 | 35.43 | Undetermined |
| S2 | M4-13 | Pen 4 | random | autumn | 61 | 20 | 20.06 | 32.76 | Undetermined |
| S2 | M4-3 | Pen 4 | random | autumn | 64 | 18 | 20.18 | 35.65 | Undetermined |
| S2 | M4-1 | Pen 4 | random | autumn | 63 | 17 | 20.34 | 25.83 | Undetermined |
| S2 | M4-30 | Pen 4 | random | autumn | 61 | 17 | 20.54 | 34.82 | Undetermined |
| S2 | M4-25 | Pen 4 | random | autumn | 46 | 17 | 20.56 | 20.88 | Undetermined |
| S2 | M4-11 | Pen 4 | random | autumn | 57 | 16 | 20.66 | 32.25 | Undetermined |
| S2 | M4-29 | Pen 4 | random | autumn | 48 | 16 | 20.71 | 29.49 | Undetermined |
| S2 | M4-16 | Pen 4 | random | autumn | 74 | 18 | 20.85 | 34.69 | Undetermined |
| S2 | M4-26 | Pen 4 | random | autumn | 58 | 17 | 21.44 | Undetermined | Undetermined |
| S2 | M4-17 | Pen 4 | random | autumn | 71 | 17 | 21.47 | 33.90 | Undetermined |
| S2 | M4-18 | Pen 4 | random | autumn | 53 | 16 | 21.79 | 35.57 | Undetermined |
| S2 | M4-27 | Pen 4 | random | autumn | 59 | 18 | 21.84 | Undetermined | Undetermined |
| S2 | M4-22 | Pen 4 | random | autumn | 60 | 17 | 22.17 | Undetermined | Undetermined |
| S2 | M4-21 | Pen 4 | random | autumn | 51 | 16 | 22.43 | 34.94 | Undetermined |
| S2 | M4-6 | Pen 4 | random | autumn | 82 | 17 | 22.46 | 32.60 | Undetermined |
| S2 | M4-15 | Pen 4 | random | autumn | 69 | 18 | 22.57 | 35.83 | Undetermined |
| S2 | M4-5 | Pen 4 | random | autumn | 76 | 18 | 22.60 | 34.86 | Undetermined |
| S2 | M4-23 | Pen 4 | random | autumn | 96 | 19 | 23.08 | Undetermined | Undetermined |
| S2 | M4-4 | Pen 4 | random | autumn | 65 | 17 | 23.23 | 35.11 | Undetermined |
| S2 | M4-19 | Pen 4 | random | autumn | 74 | 18 | 23.39 | Undetermined | Undetermined |
| S2 | M4-20 | Pen 4 | random | autumn | 68 | 17 | 23.85 | 35.20 | Undetermined |
| S2 | M4-24 | Pen 4 | random | autumn | 75 | 18 | 24.09 | 36.41 | Undetermined |
| S2 | M4-8 | Pen 4 | random | autumn | 80 | 18 | 24.41 | Undetermined | Undetermined |
| S2 | M4-31 | Pen 4 | mortality | autumn | 51 | 15 | 21.60 | 29.87 | Undetermined |
| S2 | M4-35 | Pen 4 | mortality | autumn | 49 | 16 | 21.13 | 26.25 | Undetermined |
| S2 | M4-33 | Pen 4 | mortality | autumn | 47 | 16 | 21.96 | 24.92 | Undetermined |
| S2 | M4-34 | Pen 4 | mortality | autumn | 57 | 17 | 18.25 | 24.27 | Undetermined |
| S2 | M4-32 | Pen 4 | mortality | autumn | 61 | 17 | 23.60 | 17.67 | Undetermined |
| S2 | M5-1 | Pen 5 | random | autumn | 71 | 19 | 18.98 | 35.07 | Undetermined |
| S2 | M5-5 | Pen 5 | random | autumn | 98 | 18 | 19.40 | 34.40 | Undetermined |
| S2 | M5-18 | Pen 5 | random | autumn | 56 | 16 | 19.60 | 31.68 | Undetermined |
| S2 | M5-13 | Pen 5 | random | autumn | 71 | 18 | 19.65 | 37.24 | Undetermined |
| S2 | M5-20 | Pen 5 | random | autumn | 88 | 18 | 20.01 | 35.54 | Undetermined |
| S2 | M5-14 | Pen 5 | random | autumn | 62 | 17 | 20.16 | 30.95 | Undetermined |
| S2 | M5-25 | Pen 5 | random | autumn | 77 | 18 | 20.23 | Undetermined | Undetermined |
| S2 | M5-21 | Pen 5 | random | autumn | 71 | 18 | 20.26 | 34.41 | Undetermined |
| S2 | M5-7 | Pen 5 | random | autumn | 108 | 19 | 20.49 | 34.46 | Undetermined |
| S2 | M5-24 | Pen 5 | random | autumn | 59 | 18 | 20.59 | 33.43 | Undetermined |
| S2 | M5-17 | Pen 5 | random | autumn | 75 | 18 | 20.59 | Undetermined | Undetermined |
| S2 | M5-12 | Pen 5 | random | autumn | 95 | 19 | 20.67 | 34.66 | Undetermined |
| S2 | M5-16 | Pen 5 | random | autumn | 63 | 16 | 20.67 | Undetermined | Undetermined |
| S2 | M5-3 | Pen 5 | random | autumn | 89 | 17 | 20.95 | 35.83 | Undetermined |
| S2 | M5-27 | Pen 5 | random | autumn | 84 | 18 | 20.95 | Undetermined | Undetermined |
| S2 | M5-29 | Pen 5 | random | autumn | 72 | 17 | 21.00 | 36.45 | Undetermined |
| S2 | M5-9 | Pen 5 | random | autumn | 64 | 16 | 21.01 | 33.28 | Undetermined |
| S2 | M5-19 | Pen 5 | random | autumn | 70 | 18 | 21.07 | Undetermined | Undetermined |
| S2 | M5-10 | Pen 5 | random | autumn | 82 | 18 | 21.10 | Undetermined | Undetermined |
| S2 | M5-15 | Pen 5 | random | autumn | 70 | 18 | 21.47 | 35.55 | Undetermined |
| S2 | M5-4 | Pen 5 | random | autumn | 58 | 16 | 21.53 | Undetermined | Undetermined |
| S2 | M5-23 | Pen 5 | random | autumn | 73 | 17 | 21.55 | Undetermined | Undetermined |
| S2 | M5-8 | Pen 5 | random | autumn | 107 | 19 | 21.64 | Undetermined | Undetermined |
| S2 | M5-2 | Pen 5 | random | autumn | 95 | 20 | 21.81 | 35.61 | Undetermined |
| S2 | M5-26 | Pen 5 | random | autumn | 100 | 19 | 21.90 | 35.52 | Undetermined |
| S2 | M5-30 | Pen 5 | random | autumn | 112 | 20 | 21.99 | Undetermined | Undetermined |
| S2 | M5-6 | Pen 5 | random | autumn | 73 | 17 | 22.16 | Undetermined | Undetermined |
| S2 | M5-22 | Pen 5 | random | autumn | 83 | 20 | 22.67 | Undetermined | Undetermined |
| S2 | M5-11 | Pen 5 | random | autumn | 66 | 19 | 23.57 | Undetermined | Undetermined |
| S2 | M5-28 | Pen 5 | random | autumn | 96 | 18 | 24.01 | Undetermined | Undetermined |
| S2 | M5-34 | Pen 5 | mortality | autumn | 48 | 18 | 23.29 | 23.31 | Undetermined |
| S2 | M5-33 | Pen 5 | mortality | autumn | 61 | 16 | 21.77 | 22.72 | Undetermined |
| S2 | M5-32 | Pen 5 | mortality | autumn | 55 | 17 | 22.00 | 21.95 | Undetermined |
| S2 | M5-35 | Pen 5 | mortality | autumn | 60 | 18 | 24.33 | 20.30 | Undetermined |
| S2 | M5-31 | Pen 5 | mortality | autumn | 68 | 17 | 21.26 | 18.38 | Undetermined |
| S2 | M6-1 | Pen 6 | random | autumn | 72 | 18 | 18.01 | 35.03 | Undetermined |
| S2 | M6-6 | Pen 6 | random | autumn | 60 | 16 | 19.81 | 33.31 | Undetermined |
| S2 | M6-19 | Pen 6 | random | autumn | 64 | 17 | 20.99 | Undetermined | Undetermined |
| S2 | M6-25 | Pen 6 | random | autumn | 76 | 18 | 21.00 | 36.42 | Undetermined |
| S2 | M6-7 | Pen 6 | random | autumn | 74 | 18 | 21.63 | 35.24 | Undetermined |
| S2 | M6-21 | Pen 6 | random | autumn | 75 | 17 | 21.81 | 37.98 | Undetermined |
| S2 | M6-16 | Pen 6 | random | autumn | 68 | 17 | 22.05 | Undetermined | Undetermined |
| S2 | M6-30 | Pen 6 | random | autumn | 63 | 17 | 22.16 | Undetermined | Undetermined |
| S2 | M6-29 | Pen 6 | random | autumn | 68 | 18 | 22.28 | Undetermined | Undetermined |
| S2 | M6-18 | Pen 6 | random | autumn | 66 | 16 | 22.49 | 37.41 | Undetermined |
| S2 | M6-5 | Pen 6 | random | autumn | 55 | 16 | 22.54 | 29.33 | Undetermined |
| S2 | M6-9 | Pen 6 | random | autumn | 72 | 17 | 22.75 | 37.37 | Undetermined |
| S2 | M6-2 | Pen 6 | random | autumn | 68 | 18 | 22.87 | 37.70 | Undetermined |
| S2 | M6-3 | Pen 6 | random | autumn | 60 | 17 | 23.18 | Undetermined | Undetermined |
| S2 | M6-20 | Pen 6 | random | autumn | 72 | 18 | 23.46 | Undetermined | Undetermined |
| S2 | M6-26 | Pen 6 | random | autumn | 83 | 18 | 23.55 | Undetermined | Undetermined |
| S2 | M6-23 | Pen 6 | random | autumn | 66 | 17 | 23.95 | 38.49 | Undetermined |
| S2 | M6-12 | Pen 6 | random | autumn | 67 | 17 | 23.96 | Undetermined | Undetermined |
| S2 | M6-13 | Pen 6 | random | autumn | 75 | 17 | 24.03 | 37.92 | Undetermined |
| S2 | M6-15 | Pen 6 | random | autumn | 73 | 17 | 24.62 | 36.40 | Undetermined |
| S2 | M6-22 | Pen 6 | random | autumn | 91 | 18 | 24.71 | 36.78 | Undetermined |
| S2 | M6-10 | Pen 6 | random | autumn | 64 | 17 | 24.81 | 35.43 | Undetermined |
| S2 | M6-27 | Pen 6 | random | autumn | 58 | 16 | 25.10 | Undetermined | Undetermined |
| S2 | M6-14 | Pen 6 | random | autumn | 75 | 17 | 25.70 | Undetermined | Undetermined |
| S2 | M6-4 | Pen 6 | random | autumn | 65 | 17 | 25.80 | 38.02 | Undetermined |
| S2 | M6-11 | Pen 6 | random | autumn | 85 | 17 | 25.84 | Undetermined | Undetermined |
| S2 | M6-31 | Pen 6 | mortality | autumn | 57 | 16 | 25.47 | 26.18 | Undetermined |
| S2 | M6-28 | Pen 6 | random | autumn | 98 | 19 | 26.26 | Undetermined | Undetermined |
| S2 | M6-8 | Pen 6 | random | autumn | 74 | 16 | 26.30 | Undetermined | Undetermined |
| S2 | M6-33 | Pen 6 | mortality | autumn | 56 | 16 | 22.25 | 24.05 | Undetermined |
| S2 | M6-17 | Pen 6 | random | autumn | 59 | 16 | 26.85 | Undetermined | Undetermined |
| S2 | M6-34 | Pen 6 | mortality | autumn | 67 | 17 | 28.48 | 22.44 | Undetermined |
| S2 | M6-24 | Pen 6 | random | autumn | 78 | 18 | 27.00 | Undetermined | Undetermined |
| S2 | M6-32 | Pen 6 | mortality | autumn | 54 | 16 | 24.63 | 20.20 | Undetermined |
| S2 | M6-35 | Pen 6 | mortality | autumn | 79 | 18 | 26.99 | 18.22 | Undetermined |
| S2 | M7-17 | Pen 7 | random | autumn | 73 | 18 | 19.40 | 33.86 | Undetermined |
| S2 | M7-25 | Pen 7 | random | autumn | 71 | 18 | 19.67 | 34.02 | Undetermined |
| S2 | M7-29 | Pen 7 | random | autumn | 56 | 18 | 20.14 | 29.79 | Undetermined |
| S2 | M7-27 | Pen 7 | random | autumn | 72 | 17 | 20.18 | 30.91 | Undetermined |
| S2 | M7-16 | Pen 7 | random | autumn | 70 | 17 | 20.40 | 31.89 | Undetermined |
| S2 | M7-18 | Pen 7 | random | autumn | 58 | 18 | 20.96 | 33.85 | Undetermined |
| S2 | M7-11 | Pen 7 | random | autumn | 89 | 18 | 20.98 | 33.33 | Undetermined |
| S2 | M7-26 | Pen 7 | random | autumn | 46 | 18 | 21.07 | Undetermined | Undetermined |
| S2 | M7-28 | Pen 7 | random | autumn | 64 | 19 | 21.38 | 33.85 | Undetermined |
| S2 | M7-22 | Pen 7 | random | autumn | 67 | 19 | 21.38 | 36.54 | Undetermined |
| S2 | M7-23 | Pen 7 | random | autumn | 76 | 19 | 22.14 | 36.53 | Undetermined |
| S2 | M7-20 | Pen 7 | random | autumn | 58 | 17 | 22.16 | 35.17 | Undetermined |
| S2 | M7-5 | Pen 7 | random | autumn | 61 | 19 | 22.34 | 31.80 | Undetermined |
| S2 | M7-12 | Pen 7 | random | autumn | 82 | 19 | 22.62 | 30.52 | Undetermined |
| S2 | M7-4 | Pen 7 | random | autumn | 66 | 18 | 22.70 | 32.94 | Undetermined |
| S2 | M7-7 | Pen 7 | random | autumn | 64 | 17 | 23.48 | Undetermined | Undetermined |
| S2 | M7-19 | Pen 7 | random | autumn | 62 | 17 | 23.57 | Undetermined | Undetermined |
| S2 | M7-6 | Pen 7 | random | autumn | 69 | 19 | 24.04 | Undetermined | Undetermined |
| S2 | M7-34 | Pen 7 | mortality | autumn | 65 | 19 | 25.86 | 30.22 | Undetermined |
| S2 | M7-3 | Pen 7 | random | autumn | 45 | 15 | 24.50 | 23.62 | Undetermined |
| S2 | M7-10 | Pen 7 | random | autumn | 62 | 19 | 24.53 | 35.39 | Undetermined |
| S2 | M7-15 | Pen 7 | random | autumn | 40 | 16 | 24.59 | 36.36 | Undetermined |
| S2 | M7-24 | Pen 7 | random | autumn | 82 | 19 | 24.63 | 36.30 | Undetermined |
| S2 | M7-13 | Pen 7 | random | autumn | 68 | 17 | 24.91 | 34.53 | Undetermined |
| S2 | M7-2 | Pen 7 | random | autumn | 80 | 17 | 24.91 | 33.38 | Undetermined |
| S2 | M7-31 | Pen 7 | mortality | autumn | 56 | 18 | 26.86 | 28.52 | Undetermined |
| S2 | M7-8 | Pen 7 | random | autumn | 56 | 17 | 25.79 | 35.03 | Undetermined |
| S2 | M7-1 | Pen 7 | random | autumn | 75 | 16 | 25.87 | Undetermined | Undetermined |
| S2 | M7-21 | Pen 7 | random | autumn | 60 | 17 | 25.99 | Undetermined | Undetermined |
| S2 | M7-30 | Pen 7 | random | autumn | 54 | 18 | 26.13 | 36.24 | Undetermined |
| S2 | M7-35 | Pen 7 | mortality | autumn | 61 | 19 | 24.04 | 25.04 | Undetermined |
| S2 | M7-32 | Pen 7 | mortality | autumn | 61 | 19 | 23.32 | 21.32 | Undetermined |
| S2 | M7-33 | Pen 7 | mortality | autumn | 55 | 18 | 24.47 | 20.69 | Undetermined |
| S2 | M7-14 | Pen 7 | random | autumn | 57 | 17 | 27.07 | Undetermined | Undetermined |
| S2 | M7-9 | Pen 7 | random | autumn | 66 | 18 | 28.26 | Undetermined | Undetermined |
| S2 | M8-14 | Pen 8 | random | autumn | 63 | 18 | 18.10 | Undetermined | Undetermined |
| S2 | M8-26 | Pen 8 | random | autumn | 62 | 16 | 18.57 | 27.65 | Undetermined |
| S2 | M8-16 | Pen 8 | random | autumn | 61 | 16 | 18.60 | 29.95 | Undetermined |
| S2 | M8-13 | Pen 8 | random | autumn | 72 | 17 | 18.81 | 30.22 | Undetermined |
| S2 | M8-12 | Pen 8 | random | autumn | 76 | 18 | 18.91 | 29.90 | Undetermined |
| S2 | M8-11 | Pen 8 | random | autumn | 76 | 19 | 19.06 | 28.00 | Undetermined |
| S2 | M8-10 | Pen 8 | random | autumn | 72 | 19 | 19.08 | 29.01 | Undetermined |
| S2 | M8-6 | Pen 8 | random | autumn | 72 | 18 | 19.31 | 29.26 | Undetermined |
| S2 | M8-15 | Pen 8 | random | autumn | 72 | 16 | 19.35 | 29.92 | Undetermined |
| S2 | M8-27 | Pen 8 | random | autumn | 65 | 18 | 19.68 | 29.40 | Undetermined |
| S2 | M8-20 | Pen 8 | random | autumn | 70 | 17 | 19.70 | 29.77 | Undetermined |
| S2 | M8-28 | Pen 8 | random | autumn | 101 | 19 | 19.73 | 34.22 | Undetermined |
| S2 | M8-17 | Pen 8 | random | autumn | 84 | 19 | 19.86 | 31.85 | Undetermined |
| S2 | M8-21 | Pen 8 | random | autumn | 76 | 18 | 20.20 | 31.27 | Undetermined |
| S2 | M8-25 | Pen 8 | random | autumn | 89 | 19 | 20.41 | 33.28 | Undetermined |
| S2 | M8-19 | Pen 8 | random | autumn | 78 | 16 | 20.49 | 32.74 | Undetermined |
| S2 | M8-24 | Pen 8 | random | autumn | 69 | 17 | 20.62 | 32.96 | Undetermined |
| S2 | M8-18 | Pen 8 | random | autumn | 64 | 17 | 20.67 | 32.16 | Undetermined |
| S2 | M8-5 | Pen 8 | random | autumn | 49 | 16 | 20.78 | 31.58 | Undetermined |
| S2 | M8-29 | Pen 8 | random | autumn | 64 | 17 | 20.79 | 29.82 | Undetermined |
| S2 | M8-9 | Pen 8 | random | autumn | 80 | 19 | 20.80 | 32.18 | Undetermined |
| S2 | M8-30 | Pen 8 | random | autumn | 65 | 17 | 20.82 | 31.31 | Undetermined |
| S2 | M8-3 | Pen 8 | random | autumn | 66 | 17 | 20.88 | 31.12 | Undetermined |
| S2 | M8-7 | Pen 8 | random | autumn | 69 | 18 | 21.39 | 34.88 | Undetermined |
| S2 | M8-22 | Pen 8 | random | autumn | 69 | 18 | 21.41 | 35.00 | Undetermined |
| S2 | M8-8 | Pen 8 | random | autumn | 64 | 18 | 22.08 | 33.50 | Undetermined |
| S2 | M8-23 | Pen 8 | random | autumn | 76 | 18 | 22.64 | 36.22 | Undetermined |
| S2 | M8-4 | Pen 8 | random | autumn | 66 | 17 | 23.00 | 34.51 | Undetermined |
| S2 | M8-2 | Pen 8 | random | autumn | 85 | 18 | 23.84 | 34.28 | Undetermined |
| S2 | M8-1 | Pen 8 | random | autumn | 85 | 18 | 24.84 | Undetermined | Undetermined |
| S2 | M8-32 | Pen 8 | mortality | autumn | 79 | 18 | 20.71 | 26.62 | Undetermined |
| S2 | M8-34 | Pen 8 | mortality | autumn | 52 | 17 | 20.72 | 26.15 | Undetermined |
| S2 | M8-35 | Pen 8 | mortality | autumn | 52 | 16 | 19.39 | 23.45 | Undetermined |
| S2 | M8-31 | Pen 8 | mortality | autumn | 48 | 15 | 20.77 | 19.36 | Undetermined |
| S2 | M8-33 | Pen 8 | mortality | autumn | 40 | 15 | 18.91 | 16.63 | Undetermined |
| S2 | M9-12 | Pen 9 | random | autumn | 61 | 17 | 17.50 | 27.08 | Undetermined |
| S2 | M9-8 | Pen 9 | random | autumn | 61 | 19 | 17.70 | 30.74 | Undetermined |
| S2 | M9-1 | Pen 9 | random | autumn | 73 | 19 | 18.00 | 28.29 | Undetermined |
| S2 | M9-18 | Pen 9 | random | autumn | 56 | 18 | 18.27 | 25.47 | Undetermined |
| S2 | M9-15 | Pen 9 | random | autumn | 90 | 20 | 18.41 | 28.23 | Undetermined |
| S2 | M9-4 | Pen 9 | random | autumn | 67 | 19 | 18.45 | 30.59 | Undetermined |
| S2 | M9-24 | Pen 9 | random | autumn | 53 | 18 | 18.46 | 28.76 | Undetermined |
| S2 | M9-25 | Pen 9 | random | autumn | 78 | 21 | 18.47 | 31.46 | Undetermined |
| S2 | M9-13 | Pen 9 | random | autumn | 86 | 19 | 18.62 | 29.38 | Undetermined |
| S2 | M9-19 | Pen 9 | random | autumn | 58 | 19 | 18.75 | 26.62 | Undetermined |
| S2 | M9-11 | Pen 9 | random | autumn | 75 | 20 | 18.75 | 24.53 | Undetermined |
| S2 | M9-6 | Pen 9 | random | autumn | 61 | 19 | 19.38 | 29.53 | Undetermined |
| S2 | M9-7 | Pen 9 | random | autumn | 50 | 19 | 19.52 | 27.55 | Undetermined |
| S2 | M9-2 | Pen 9 | random | autumn | 64 | 18 | 19.58 | Undetermined | Undetermined |
| S2 | M9-9 | Pen 9 | random | autumn | 56 | 18 | 19.79 | 24.79 | Undetermined |
| S2 | M9-29 | Pen 9 | random | autumn | 77 | 20 | 19.93 | 32.05 | Undetermined |
| S2 | M9-14 | Pen 9 | random | autumn | 90 | 118 | 20.33 | 28.76 | Undetermined |
| S2 | M9-17 | Pen 9 | random | autumn | 76 | 19 | 20.82 | 25.99 | Undetermined |
| S2 | M9-23 | Pen 9 | random | autumn | 76 | 18 | 21.13 | 29.17 | Undetermined |
| S2 | M9-26 | Pen 9 | random | autumn | 66 | 19 | 21.20 | 32.13 | Undetermined |
| S2 | M9-5 | Pen 9 | random | autumn | 55 | 17 | 21.35 | 29.40 | Undetermined |
| S2 | M9-20 | Pen 9 | random | autumn | 54 | 17 | 21.63 | 19.90 | Undetermined |
| S2 | M9-10 | Pen 9 | random | autumn | 80 | 20 | 21.69 | 33.33 | Undetermined |
| S2 | M9-3 | Pen 9 | random | autumn | 80 | 20 | 21.70 | 33.41 | Undetermined |
| S2 | M9-16 | Pen 9 | random | autumn | 75 | 19 | 21.77 | 32.41 | Undetermined |
| S2 | M9-27 | Pen 9 | random | autumn | 45 | 16 | 21.78 | 25.33 | Undetermined |
| S2 | M9-21 | Pen 9 | random | autumn | 73 | 19 | 21.99 | 32.55 | Undetermined |
| S2 | M9-22 | Pen 9 | random | autumn | 60 | 18 | 22.14 | 30.77 | Undetermined |
| S2 | M9-28 | Pen 9 | random | autumn | 99 | 21 | 22.20 | 35.48 | Undetermined |
| S2 | M9-30 | Pen 9 | random | autumn | 75 | 20 | 22.44 | 35.80 | Undetermined |
| S2 | M9-32 | Pen 9 | mortality | autumn | 60 | 16 | 22.43 | 27.12 | Undetermined |
| S2 | M9-31 | Pen 9 | mortality | autumn | 71 | 21 | 20.12 | 26.40 | Undetermined |
| S2 | M9-35 | Pen 9 | mortality | autumn | 64 | 19 | 19.51 | 24.30 | Undetermined |
| S2 | M9-33 | Pen 9 | mortality | autumn | 75 | 19 | 22.67 | 21.40 | Undetermined |
| S2 | M9-34 | Pen 9 | mortality | autumn | 53 | 17 | 19.77 | 18.24 | Undetermined |
